# Historical case study of a transfer experiment that demonstrated the importance of the estuarine ecosystem for the survival of Chinook salmon (*Oncorhynchus tshawytscha*) at the Campbell River estuary, British Columbia, Canada

**DOI:** 10.1111/jfb.70443

**Published:** 2026-05-20

**Authors:** Colin D. Levings, J. Steve Macdonald

**Affiliations:** ^1^ Scientist Emeriti, Fisheries and Oceans Canada, Pacific Science Enterprise Centre West Vancouver British Columbia Canada

**Keywords:** Chinook salmon, ecosystem, estuary, smolt‐to‐adult survival, transfer experiment

## Abstract

Estuary dependence to the survival of Chinook salmon was investigated at the Campbell River estuary, British Columbia, Canada. Replicate batches of marked smolts were transferred from a hatchery and released in 1983, 1984 and 1985 at four ecosystems, two that ensured estuarine experience (river, estuary) and two seawards of the estuary (transition, marine). Juveniles were tracked with beach seine collections, providing residency times in the four ecosystems. Marked adult salmon were enumerated in the fishery and spawning grounds for 6 years. Recoveries from the marine and transition ecosystem releases comprised 35.1% of the total recoveries (smolt‐to‐adult survival 0.34%), whereas 64.9% were from river and estuary ecosystems (smolt‐to‐adult survival 0.60%). Fish released in the marine ecosystem in 1985 were recovered at higher rates than in 1983 or 1984, creating a statistical interaction between year and release location that implicated differences in annual ocean conditions. Results are related to present‐day salmon conservation challenges and restoration consequences. In essence, transfer experiments complement other approaches to studies of salmon estuary dependence but require considerable field labour efforts and attention to temporal replication to achieve statistical rigour. Experimental interpretation can be improved with investigations expanded to include several populations and estuaries and a more extensive consideration of annual variability in river and ocean conditions.

## 1 INTRODUCTION AND CONTEXT

In this paper, we provide the case study narrative and complete results and analysis of a juvenile Chinook salmon transfer experiment designed to try and directly assess the effect of the estuary ecosystem on smolt‐to‐adult survival. Conducted in the early 1980s at the Campbell River estuary, British Columbia, Canada, it was initiated when managers identified a need for a straightforward approach to collect stronger inferential data on how the estuary affected smolt‐to‐adult survival than those provided by small‐scale and detailed ecosystem studies. There were very limited data available on estuary effects at this time in the history of Chinook salmon ecology. A seminal paper at the time by Reimers (1973) in Oregon showed that fish with a growth check from estuarine residency as juveniles survived better than those in which it was absent. Data on estuary effects on survival were not available for any system in British Columbia, but there was an increasing demand for information by regulators, conservationists and restoration ecologists.

Chinook salmon (*Oncorhynchus tshawytscha*) range from Monterey Bay to the Chukchi Sea in the northeast Pacific (Hecht et al., 2015). They are the largest of the six species in the genus *Oncorhynchus* and are a very important salmon species on the West Coast of North America for both First Nations and settlers (Atlas et al., 2021). For example, early‐run Chinook salmon in the Fraser River in British Columbia, Canada, are important for late‐winter food for the Halq'eméylem First Nation. Chinook salmon are also highly valued by commercial and sport fishers. However, the annual abundance of Chinook salmon is now frequently well below historical levels. Many stocks are considered endangered or threatened and no longer support fisheries in Canada and the USA (Welch et al., 2021). Chinook salmon are also key elements in coastal ecosystems and food webs. They are prey for apex predators such as killer whales from Alaska to California (Van Cise et al., 2024) and are important as lower‐level consumers in pelagic (e.g., Lerner & Hunt, 2024) and estuarine ecosystems (e.g., Davis et al., 2024) along the northeast Pacific coast.

Accrued over about the past 50 years, a large body of literature supports the idea that Chinook salmon fry and smolt fitness is linked to estuary ecosystems (summarized in Levings, 2016; Quinn, 2018). Briefly, the juvenile fish achieve a growth benefit via the estuary detrital food web (Healey, 1982; Kennedy et al., 2018; Macdonald et al., 1987), avoid predation by utilizing estuary habitat such as marsh vegetation (Chalifour et al., 2021; Gregory & Levings, 1996; Simenstad et al., 2000) and complete osmoregulatory readiness to ocean salinity (Clarke & Shelbourn, 1985; Houde et al., 2019). These findings have led to agency estuary protection policies (e.g., Anon, 2023) and citizen conservation efforts (e.g., MacDuffee et al., 2023). Because of the substantial loss of estuary habitat along the West Coast of North America [e.g., US estuaries, 86% loss (Brophy et al., 2019); Fraser River estuary, >70% loss (Kehoe et al., 2021)], there is also a substantial history of restoration efforts ranging from habitat reconnections (Tutty et al., 1983; Diefenderfer et al., 2021), marsh transplants (Levings & Macdonald, 1991; Stewart et al., 2024) to rip rap removal (Des Roches et al., 2024).

### 1.1 Background – Experiment development and reporting status

When we began our Campbell River work in 1981, our belief in estuary importance to survival was based largely on their length of utilization of specific habitats by life stages of each species. The concept of a direct case study approach using transfer experiments to assess smolt‐to‐adult salmon survival relative to the exclusion of ecosystem conditions in estuaries was not well developed. A notable paper on the topic was given by the late Dr. Marty Kjelson at a 1981 conference and published in Kjelson et al. (1982). The paper compared the smolt‐to‐adult survival of hatchery Chinook salmon released into the degraded upstream reaches of the Sacramento River in California with those released into the Sacramento River estuary. Fish released into the estuary survived better than the river‐released fish, and the work was later expanded to include additional river–estuary release experiments (reported in Kjelson & Brandes, 1989). Recognizing the value of this direct approach, we adopted the same release, or transfer, plan and released batches of specifically marked Chinook salmon into four ecosystems – river, estuary, transition and marine – at the Campbell River estuary and adjacent coastal area. The California work primarily investigated temperature and flow conditions as biophysical variables affecting survival. We took a comprehensive ecological approach and accompanied our work with a strong ecosystem and habitat programme, including residency and physiology studies (e.g., Macdonald et al., 1988), diet analyses (Kask et al., 1986), salt wedge surveys (Macdonald et al., 1987), wild and hatchery fish interaction studies (Levings et al., 1986) and comprehensive beach seine surveys to assess abundance of all salmon species (Brown et al., 1987).

Preliminary results and interpretations of the survival of the transferred fish were presented earlier as we awaited all returning tagged fish (Levings, 2016; Levings et al., 1989; Macdonald et al., 1988). Of these, the 1988 paper provided the most detailed interpretation of the recoveries but was based on only 163 adults; our 1989 paper was based on 638 adult recoveries and dealt primarily with the distribution of marked adult fish in the fishery. The results in Levings (2016) did not include any updated interpretation. We believe it is a responsibility to publish the full results of this major experiment now with all 1903 adult recoveries available for analysis and interpretation. The final synthesis of data from all adult recoveries complements our current understanding of the importance of an estuary to the species and fosters continued ecosystem management in estuaries. It also highlights the value of long‐term studies at larger experimental scales when examining life‐history stages and the contemporaneous environmental factors that limit Chinook survival.

### 1.2 The null hypothesis

We manipulated the natural seaward migration of four groups of juvenile Chinook salmon such that two of the groups were released outside of the estuary to determine if estuary deprivation affected survival to adults. Our null hypothesis, ‘Estuarine residency does not promote Chinook salmon survival’ (Levings, 1984) is at a scale and complexity that limits practical opportunities for extensive replication and therefore also limits extrapolation of results in a broader geographical context to include more Chinook populations. This is common to the case study approach but does provide the opportunity for meta‐analyses with other whole ecosystem examinations (e.g., Bugnot et al., 2024). We reference other experimental approaches, making comparisons and recommendations for future whole ecosystem transfer experiments to help researchers investigate the complex factors involved in design and execution of future studies. We feel that the ‘lesson learned or adaptive learning’ aspect of the work is a major benefit of reporting on the project. At the very least, the experiment illustrates estuarine influence on a single stock of Chinook salmon on a once‐productive and iconic river system, known internationally by conservationists (Haig‐Brown, 1946) and recreational anglers (Egan, 1988).

## 2 METHODS

There was no requirement for an ethics board approval or formal animal care guidelines for field collections when the animals were released for this study 43 years ago.

Experimental release locations and recoveries of smolts and adults.

Details on the methods used in the marking and transport of the experimental Chinook salmon smolts, and the release and recovery locations, including past analyses, are provided by Levings et al. (1984), Kotyk et al. (1985) and Macdonald et al. (1988). Recovery methods for both adults and juveniles and sampling equipment details are described in Supporting Information File A and Kotyk et al. (1985). A brief overview is provided below.

**FIGURE 1:**
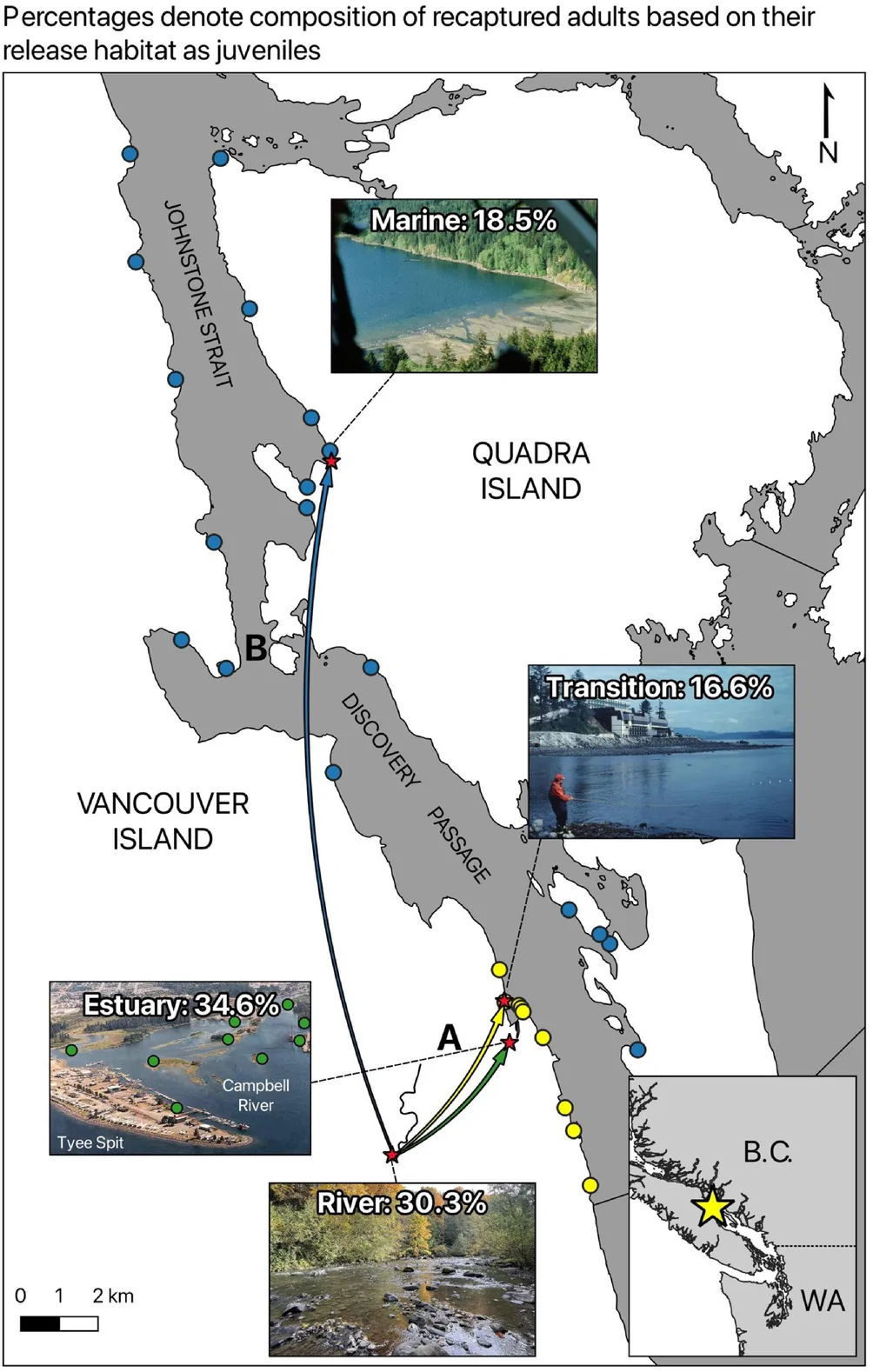
Map of the study area showing the river, estuary, transition and marine ecosystems with photos, where the marked Chinook salmon smolts were released (red stars). Coloured dots mark locations within ecosystems where beach seine collections monitored juvenile migration before and for 8 months following releases in 1983, 1984 and 1985. Detached images of each release ecosystem at ends of the arrows are accompanied with the percentage of the total adult recovery from fisheries, hatcheries and on the spawning grounds (*n* = 1903) in the years following release. Symbols: blue ○ – marine ecosystem; green ○ – estuary ecosystem; yellow ○ – transition ecosystem. (A) Campbell River; (B)) Seymour Narrows. Photo credits: Quinsam River – All trails public site; estuary – 1998 photo from Dawe et al., 2000 with permission; transition and marine – Levings collection.

The Campbell River and its main tributary, the Quinsam River, have riparian shorelines with deciduous trees (Figure 1) below a hydroelectric facility that provides a combined annual flow of 108 cm (baseline, as the Campbell River has a hydroelectric facility with controlled flows). During our study, the eastern sector of the Campbell River estuary (area approximately 75 ha, Figure 1) was characterized by a mixture of natural marsh and transplanted marsh on artificial islands (see Levings & Macdonald, 1991), and the western sector was used for log storage and a small vessel marina. The group of experimental fish destined for the Quinsam River ecosystem was released immediately below the hatchery, about 5 km upstream from the estuary, and those destined for the estuary ecosystem were released approximately 1 km from the mouth of the river, near the upstream extent of tidal influence. The transition ecosystem was defined as the area of the gravel/sand at the mouth of the estuary along the marine foreshore on the south side of Discovery Passage where reduced salinity was measured. A ferry terminal, foreshore developments and a marina were located in the eastern sector of the transition ecosystem (Figure 1). Fish were released to this ecosystem near the end of Tyee Spit. The marine ecosystem was defined by 8 km of gravel and rocky shorelines on Johnstone Strait and Discovery Passage. Locally, this seaway is characterized by steep, narrow intertidal zones, with the turbulent Seymour Narrows to the northwest and Discovery Passage to the southeast. The fish were released at the inner extent of Deepwater Bay near a well‐protected and extensive sand and rocky beach on the southern reach of Johnstone Strait (Figure 1). As the smolts moved out of our study area, northwest through Johnstone Strait to the coastal ocean (Supporting Information File B), they were exposed to the same nearshore biophysical conditions in what we call our marine ecosystem, although less so as they grow larger and use deeper water. Both the transition and marine ecosystem shorelines are affected by swift tidal currents (maxima up to 60 cms), necessitating release into an embayment outside the influence of the strongest currents. Comprehensive descriptions of the estuary and the Johnstone Strait Discovery Passage area at the time of release, including shoreline types and oceanography, are given in Thomson (1981), Bell and Thompson (1977), Levings et al. (1986) and Macdonald et al. (1988). A collation of physical and biological parameters in each of the release ecosystems was created to consider conditions that may have influenced annual variability in the adult recoveries (Table 1).

**TABLE 1:** Selected biological, physical and chemical characteristics of the four ecosystems (river, estuary, transition and marine) where the experimental Chinook salmon smolts were released.

Ecosystem/parameter	River	Estuary	Transition	Marine
Area (km^a^)^b^	0.09	0.45	0.04	45
Quinsam River discharge^a^ (m^c^•s^−1^)	1983: <10 1984: <10 1985: < 4	n.a.	n.a.	n.a.
Campbell River discharge^a^ (m^c^•s^−1^)	1983: <115 (336/225)^c^ 1984: <115 1985: <60 (115)^c^	n.a.	n.a.	n.a.
				
Water surface temperature (°C) on release date^d^	11.8	10.2	10.8	9.2
				
Annual mean water surface temperature (°C)				
1983	13.8^e^	11.8^f^	11.6^f^	11.3^f^
1984	14.0	12.2	11.5	10.7
1985	15.0	12.9	11.6	11.5
Surface salinity (psu) annual range^g^	0	0–4.57 (2.9)^h^	4.1–28.6 (11.7)^h^	28.6–31.6 (33.3)^h^
Potential predators on Chinook salmon smolts^i^, ^j^	Birds Mergansers (*Mergus serrator*); Cutthroat trout (*Oncorhynchus clarki*)	Harbour seals *(Phoca vitulina richardi)*; Herring and Bonapare gulls (*Larus argentatus* and *Larus philadelphia*) Mergansers Bald eagles (*Haliaeetus leucocephalus*) Coho salmon smolts Cutthroat trout	Bonaparte gulls Mergansers Seals Herring gulls Cutthroat trout	Bonaparte and Herring gulls Buffalo sculpin *(Enophrys bison)* Dolly Varden char (*Salvelinus malma*)
Hatchery Chinook smolt abundance (number per beach seine haul)^k^	nd	1983 – 5 1984 – 5 1985 – 28	1983 – 12 1984 – 17 1985 – 36	1983 – 1 1984 – 3 1986 – 14
All juvenile salmon abundance (number per beach seine haul)^k^	nd	1983 – 22 1984 – 25 1985 – 96	1983 – 48 1984 – 75 1985 – 100	1983 – 38 1984 – 110 1985 – 215

*Note*: See footnotes for data sources.

Abbreviations: n.a., not applicable; n.d., data not available.

^a^ May–August of each year.

^b^ Transition and marine ecosystem areas include the reach sectors bounded by their symbols to the north and south and to middle of Discovery Passage. The river and estuary ecosystems are bounded by channel width and length and the low salinity area behind Tyee Spit.

^c^ Indicates spill events in early June and early July 1983 and early June 1985. No spill events in 1984.

^d^ Mean temperatures at the sites over years (1983–1985) on the day the fish were released (from Macdonald et al., 1988).

^e^ Mean river temperature for river ecosystem for April–August from Blackmun et al. (1985) for 1983, from Lukyn et al. (1985) for 1984 and extrapolated from Campbell River airport air temperatures for 1985.

^f^ Mean estuary, transition and marine ecosystem water temperatures for April–August for each release year by Levings et al. (in prep.)

^g^ Representative annual salinity ranges (1983 data from Kask et al., 1988).

^h^ Mean salinities at the release sites over years (1983–1985) on the day the fish were released (from Macdonald et al., 1988).

^i^ Macdonald et al. (1988).

^j^ Macdonald et al. (1987).

^k^ Brown et al. (1987).

Earlier studies in the Campbell estuary, before the hatchery opened, found that the Chinook salmon ocean‐type fry life‐history stage (as described by Healey, 1983), also known as the ‘Fall type’ as opposed to the ‘Spring type’, was dominant form rearing in the estuary during spring and early summer (Goodman & Vroom, 1974). Later, they may remain in local sheltered marine areas through the autumn as they migrate to rear as adults in coastal waters as far north as the Gulf of Alaska (Healey, 1983). Therefore, our initial plan was to release the fish as ocean‐type fry in an effort to mimic wild fish. Ocean type are about 0.5–0.8 g (40–60 mm) during residence in the Campbell River estuary (Goodman & Vroom, 1974; Levings et al., 1986; Raymond et al., 1985). However, for operational reasons at the Quinsam hatchery, the size of fish could not be reduced by manipulating water temperature and rations during the fall and winters of 1982–1984 (Levings et al., 1989). As a result, our fish weighed between 2.5 and 3.0 g (65–70 mm length) when they were released. The length and weight range of our experimental fish did approximate the upper length and weight (56 mm, 2.8 g) of wild fry found by Goodman and Vroom (1974) and Raymond et al. (1985) during spring surveys. Our fish were considerably smaller than Quinsam River hatchery ‘production run’ Chinook salmon, which typically ranged from 5 to 6 g (Sheng, 2010), but were osmotically ready for marine conditions, having passed a saltwater challenge test before release (Clarke and Blackburn (1977).

Each year, 12 separate batches of Chinook salmon were identified with unique coded wire tag codes and an adipose clip. Mean numbers per batch by year with standard error of the mean (SEMn) were as follows: 1983 – 11,694 (SEMn – 26); 1984 – 11,782 (SEMn – 58); 1985 – 10,588 (SEMn – 57) (Supporting Information File C – Table 1). Each batch became one of three replicates released annually on 21 April 1983, 24 April 1984 and 23 April 1985 into one of four ecosystems (Figure 1: river, estuary, transition and marine). The fish were transported in canvas buckets suspended beneath a helicopter (Supporting Information File D). Each batch experienced approximately the same amount of flying time (about 15 min). The releases started on ebbing tides with a 2.8–3.5 m chart datum at about 10:00 AM and took about 2–3 h to complete. A total of 408,784 smolts were released during the 3 years of this experiment. Subsequently, adult recoveries were made through 1990 in the sport and recreational fishery, during spawning ground surveys or upon their return to the hatchery (Table 2). Recoveries were documented by the regional mark recovery programme (MRP), which annually collates tag returns from fisheries in Canada and the United States (Supporting Information Files A). The release experiments also afforded the opportunity to monitor the dispersal of juveniles each year bi‐weekly for 5 months postrelease, with intensive beach seining efforts in the estuary and locations seaward to estimate residency times of each release group as they left the experimental area (Figure 1; Supporting Information Files A and B).

**TABLE 2:** Total recoveries (in parentheses) of adult fish captured in the fishery (C) and through escapement (E) to the spawning grounds or the hatchery by year and juvenile release location.

Release year	Recoveries	River	Estuary	Transition	Marine	Probability
1983	Catch	45,56,68 (169)	74,92,75 (241)	23,26,12 (61)	14,11,15 (40)	
	Escapement	14,23,17 (54)	11,16,15 (42)	5,3,3 (11)	1,5,4 (10)	
	E/C	0.32	0.17	0.18	0.25	0.049
	Total (628)	223	283	72	50	
1984	Catch	40,28,42 (110)	70,38,70 (178)	14,4,40 (58)	13,17,17 (47)	
	Escapement	7,4,6 (17)	5,3,4 (12)	2,4,1 (7)	2,1,0 (3)	
	E/C	0.15	0.07	0.12	0.06	0.142
	Total (432)	127	190	65	50	
1985	Catch	48,80,68 (196)	59,64,45 (168)	40,36,73 (149)	69,73,80 (222)	
	Escapement	11,10,9 (30)	5,5,8 (18)	8,10,12 (30)	10,9,11 (30)	
	E/C	0.15	0.11	0.20	0.14	0.225
	Total (843)	226	186	179	252	

*Note*: Recoveries are also provided as numbers from three individual tag codes. A total of 1903 Chinook salmon were recovered. The χ^2^ probability of independence between recovery method and release location is provided in the right‐hand column to guide the test of the null hypothesis: Ho: ‘catch (C) relative to escapement (E) is consistent regardless of release location as evidence that straying was not a factor in numbers recovered’. Significance (*p* < 0.05) occurred only among the 1983 releases, when marine and transition escapement was *larger* than expected, giving confidence that fish released each year had an adequate opportunity to imprint on their natal system.

### 2.1 Analysis methods

The analyses required to summarize the results of this experiment are multifaceted and will be explained individually as follows:

- We investigated the possibility that fish released to the transition and marine sites were improperly imprinted on Campbell and Quinsam water. In this case, high rates of straying would be expected and demonstrated if river recoveries were underrepresented in escapement (E) relative to catch (C) among the groups of smolts that were denied an estuarine residency. The association between adult numbers recovered by each method (E/C ratio) and their release location as smolts was chosen as a test of straying each year (χ^2^ – *p* = 0.05). A similar analysis before all returns were available performed by Levings et al. (1989) did not detect straying.
- Survival, measured as adults captured in the recreational and commercial fisheries and returns to the hatchery and spawning grounds, was examined against the influence of two factors: release location (*n* = 4) and year of release (*n* = 3). Tag codes (*n* = 3) within the levels of each factor were originally intended as replicated treatments to assess possible interaction between the year and location of release. Fish within each tag code were thought to be genetically distinct, resulting from the fertilization of randomly chosen parents and reared in separate batches for release on separate trips to each location. We confirmed that each tag code was raised in separate troughs following hatching, but in the absence of hatchery records, we were unable to confirm the genetic independence of the tag codes. Furthermore, each tag code was treated to uniform hatchery practices designed to control environmental criteria, which further challenges the independence of the tag codes as true replicates and the representation of these fish as surrogates for natural populations.
  Two model approaches were therefore used to address our uncertainty with tag code independence:
  Model 1 evaluated the influence of the single fixed factor ‘Release Location’ (B_release_) on adult recoveries (Z _recoveries_) based on the sum of the three tag codes that were originally intended as replicates (tag code release numbers provided in Supporting Information File C). With the three experimental years providing independent replicates, tag code replication was not a design requirement, and pseudo‐replication was avoided. However, within this design (i.e., ‘Year’ unavailable as a factor) we were unable to examine ‘Year by Release Location’ interaction, which was suspected from the initial evaluation of the partial dataset (Levings et al., 1989; Macdonald et al., 1988).
  Model 1:
$$Z_{\text{recoveries}}=a+B_{\text{release}}+e.$$
  Subsequently, in Model 2, a three‐factor model with release location (*n* = 4) and year of release (*n* = 3) as fixed predictors and tag code (*n* = 3) as a third random factor was also examined. The use of a mixed‐model design followed recommendations provided by Millar and Anderson (2004) and Davies and Gray (2015) for cases where simple pseudo‐replication (Hurlbert, 1984) is suspected. As independent replicates, the tag codes would normally be used to estimate the error mean square (MS_e_) for the calculation of the F‐statistic in a two‐way fixed‐effect design. Estimates of higher‐order interaction in Model 2 were used in place of the MS_e_ in the three‐way mixed design (Zar, 1974). The value of incorporating pseudo‐replicates in models to enhance the description of complex ecosystems has been under debate since 1984 (Hurlbert, 1984 vs. Oksanen, 2001, Cottenie & De Meester, 2003) but proved useful in this study. Accordingly, the model tested the fixed treatment effects and the treatment interaction (B_release_ × D_year_) relative to the remaining error, including estimates of the highest‐order interactions with ‘Tag Code’ (C_tag_) as a random factor. The traditional two‐way design with replicate tag codes to estimate MS_e_ and Model 2, as an alternative method to partition the model's error variance, provided equivalent conclusions.
  Model 2:
$$Z_{\text{recoveries}}=a+B_{\text{release}}+C_{\mathrm{tag}}+D_{\text{year}}+B_{\text{release}}{\mathrm{xD}}_{\text{year}}+B_{\text{release}}{\mathrm{xC}}_{\mathrm{tag}}+\;D_{\text{year}}{\mathrm{xC}}_{\mathrm{tag}}+e.$$
  In both models, a covariate to adjust for the 7% difference in the mean number of smolts released among years was discarded, as it had little influence on reducing the error variance and no effect on the model outcomes. A transformation of the criterion metric [Z_recoveries_ = ln(X)] to address heterogeneity of variance among sample means and reduce other possible violations of parametric assumptions, such as non‐normal error distribution, was based on an examination of the variance–mean relationship using Taylor's Power Law (Elliott, 1977). Models were run with a general linear model routine (GLM – Minitab 2020). Statistical significance among comparisons was set at *p* = 0.05, and actual *p*‐values are provided in the text. Sources of statistical variation are provided by figures and tables.
- Collections of marked smolts from 2965 beach seines, performed bi‐weekly during a 5‐month period postrelease each year (Brown et al., 1987; Levings et al., 1984), provided residency estimates within the entire experimental area for each release group. Initially reported in Macdonald et al. (1988), in this paper, we revaluated these data to include residency estimates within each release ecosystem while providing greater resolution of movement among them. The earlier analysis involved regression analysis comparisons of declining catch sizes (catch size on each trip at each seining site as replicates) among each release group and release year as the fish left the entire experimental location (after Macdonald et al., 1988; tabular data are represented in the Results for convenience). Now, with a fresh analysis, we provide figures describing the length of time (in days between first and last capture) when 80% and 100% of the annual catch of marked smolts from each release group were captured by seine in each of the three ecosystems each year. The 80% limit estimates were meant to track the behaviour of the bulk of the fish, removing outliers. Considering 100% of the capture data provided a residency estimate for river‐ and estuary‐released fish in the marine ecosystem, where sampling effort was limited by the relative magnitude and greater depth of the sample area, which likely contributed to smaller recoveries. A proven ability to recover marked individuals from each release group throughout a protracted postrelease period gave us confidence in the consistency of the beach seine as a methodology.
- Annual age composition of the adults from fishery and hatchery recoveries were juxtaposed to the four locations they were released as juveniles to further examine the role of estuarine residence on survival. A χ^2^ test of association compared age frequency among release locations and provided estimates of mean age of recovery. Results are presented in figures.

## 3 RESULTS

**FIGURE 2:**
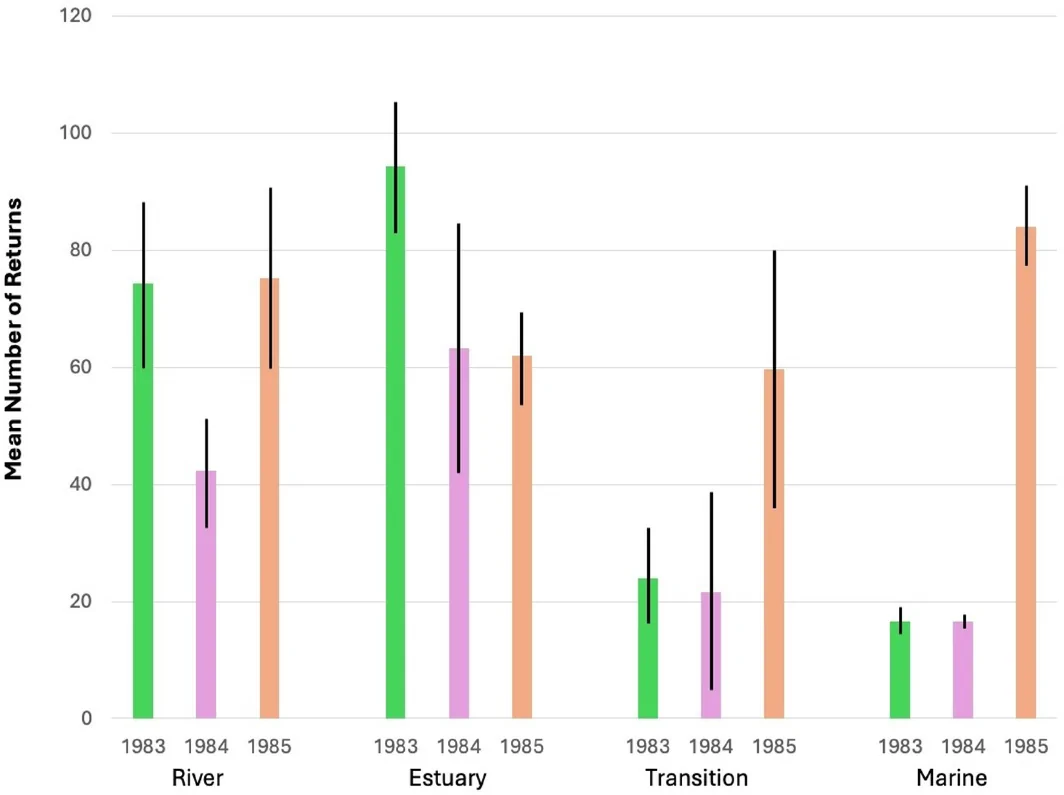
The left‐hand axis measures the mean number of adult Chinook salmon from three tag codes, recovered from hatchery and spawning ground surveys, and sport and commercial fisheries following their release in 1983 (*n* = 628 – green), 1984 (*n* = 432 – mauve) and 1985 (*n* = 843 – peach). Recoveries are further subdivided based on the ecosystem they were released as smolts: river, estuarine, transition and marine. Year‐by‐release location categories were composed of approximately 35,000 smolts in 1983 and 1984, and 32,000 in 1985. Each release was divided into three tag codes (Supporting Information File C – Table 1, smolts per year by tag code released to each ecosystem) for replication and statistical examination of the adult recoveries [analysis of variance (ANOVA), Table 3, Model 2). Vertical black bars are standard deviations of the mean number of adults recovered on the left‐hand axis. Adult recovery is also expressed as an estimate of survival of each release category each year in the Supporting Information File C – Table 1 (C/N).

Recoveries of adult Chinook salmon in the decade following the experimental releases in 1983 (628), 1984 (432) and 1985 (843) provided a total of 1903 fish recovered, following release to four locations as a test of the influence of early estuary residence on adult survival (Figure 2). There was no evidence that our experiment caused unexpected straying of returning adults, as the catch and escapement recoveries were either independent of their release location (1984 and 1985 – *p* < 0.05, Table 2) or, in 1983, the escapement recovery estimate for fish deprived of the estuary was larger than expected, whereas the high‐seas catch was smaller (*p* < 0.05).

The majority of the recoveries were from the river and estuary ecosystem release groups (576 and 659 fish, respectively; 64.9% of the total returned, Figure 1), whereas the fewest were from transition and marine releases (316 and 352 fish, respectively; 35.1% of the total). This pattern was statistically insignificant when release ecosystem was examined as the sole fixed factor with release years as replicates (Table 3, Model 1 – ‘Release Location’, *p* > 0.05). Examination using a model where the interaction between the two fixed factors was measured (with individual tag codes considered as a random factor) does demonstrate the influence of estuarine residence, but the pattern was driven by observations in 1983 and 84, not in 1985, when marine and transition release returns were larger than expected (Table 3, Model 2 – ‘Release Location by Year interaction’, *p* < 0.05). This pattern produced larger returns in 1985, and fewer in 1984 when river release returns were also depressed relative to other years (Figure 2; Table 3 – ‘Year’, *p* < 0.05). Mean percentage survival ranged from 0.14% for marine releases in 1984 to 0.79% for estuary releases in 1983, a combined survival of 0.60% versus 0.34% when contrasting fish with and without an estuarine experience (Supporting Information File C), with an overall survival of 0.47%.

**TABLE 3:** Analysis of variance (ANOVA) table resulting from the analysis of estimates of adult Chinook salmon recoveries depicted in Figure 2.

Model	Source	Effect	*df*	F‐value	*p*‐Value
1	Release location	Fixed	3	1.95	0.201
	Error		8		
	Total		11		
2	Release location	Fixed	3	42.35	0.00
	Release year	Fixed	2	12.11	0.02
	Tag code	Random	2	0.94	0.525
	Year by location		6	6.90	0.002
	Year by tag		5	1.81	0.192
	Location by tag		6	0.5	0.797
	Error		11		
	Total		35		

*Note*: Replicate groups were released to four locations (Figure 1) in each of 3 years. Equation ‘1’ describes a one‐factor model, where year is the replicate to test the influence of release location (B_release_). Equation ‘2’ describes a two‐fixed‐factor model, where D_year_ is a second factor to examine interaction. The replicate tag code (C_tag_, *n* = 3) is included as a random factor. Analysis performed using Minitab statistical software (2000) General Linear Model (GLM) procedure.

**FIGURE 3:**
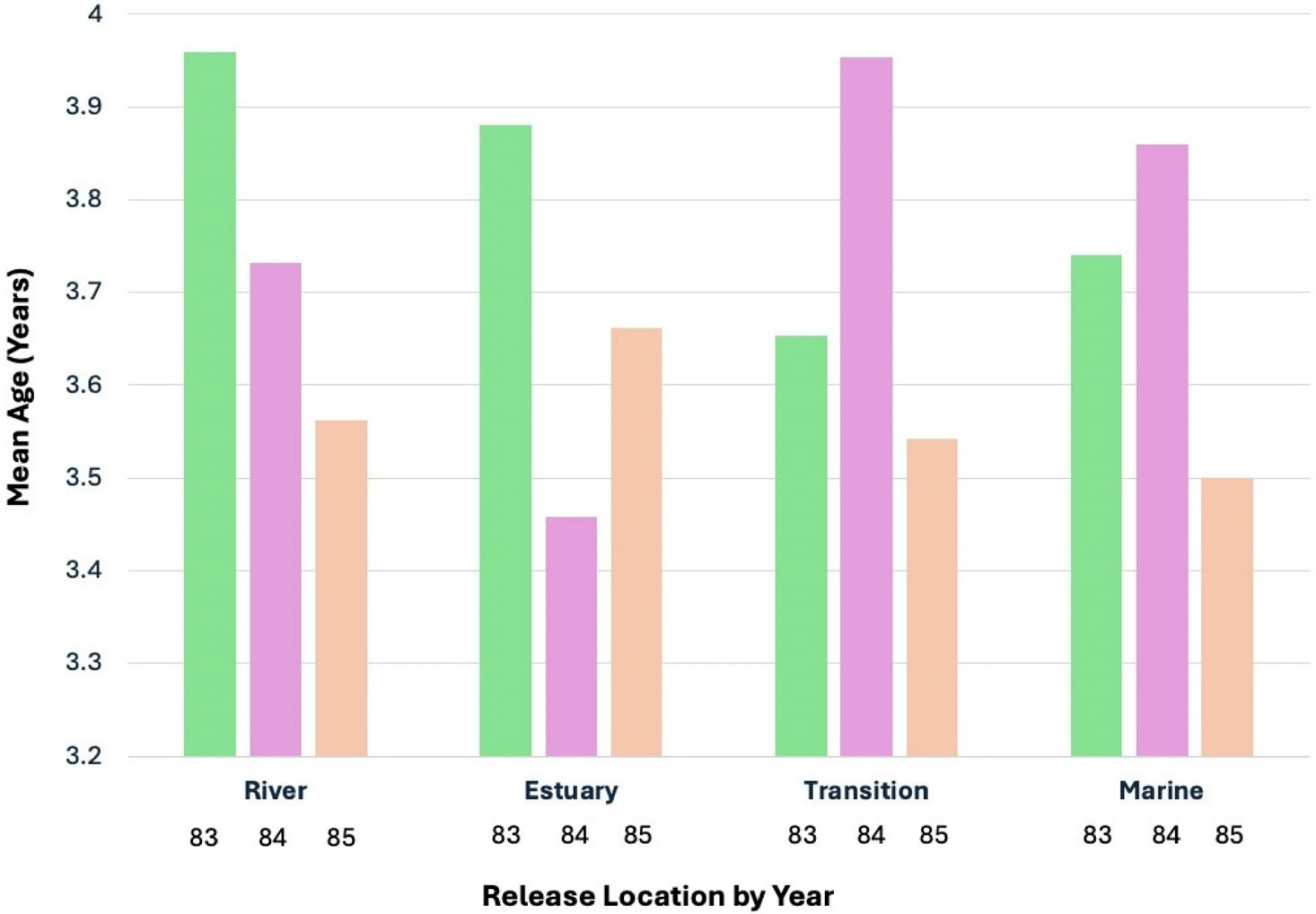
The average age of fish (years) recovered from the fishery and escapement to the natural spawning grounds and the hatchery each year (83 – green, 84 – mauve, 85 – peach) from each of the release groups.

Of the total recovered, 11.4% were 2 years old, 28.6% were 3 years old, 40.5% were 4 years old, 18.9% were 5 years old and 0.5% were 6 years old. In 1983 and 1984, but not in 1985, the release location had an influence on the age frequency of the eventual recoveries (Figure 3 – χ^2^ – *p* = 0.05). However, this influence was inconsistent among years: fish that experienced estuaries as juveniles were older at recovery in 1983 but younger in 1984 (Supporting Information File E – Figure 1).

**FIGURE 4:**
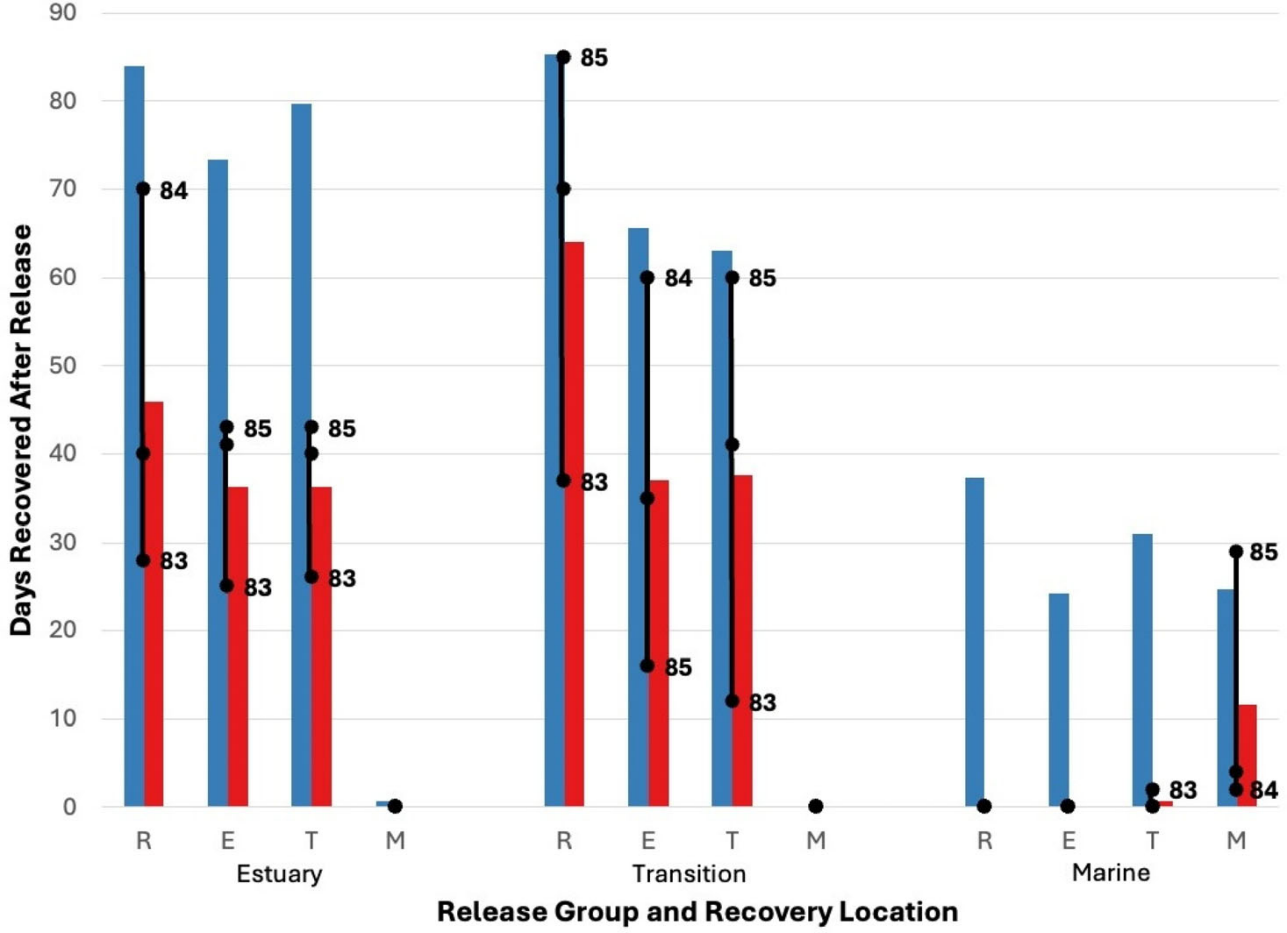
Red bars represent the number of days between initial capture and the date when 80% of the Chinook salmon smolts from each release group were recovered by beach seining in the estuary, transition or marine ecosystems, 1983–1985. Vertical lines provide annual range in residency estimates from 1983 to 1985 based on the 80% recovery estimation method of residency. Blue bars provide ancillary residency estimates using the period that 100% of the Chinook salmon smolts were recovered and therefore not limited by the small catch sizes.

**FIGURE 5:**
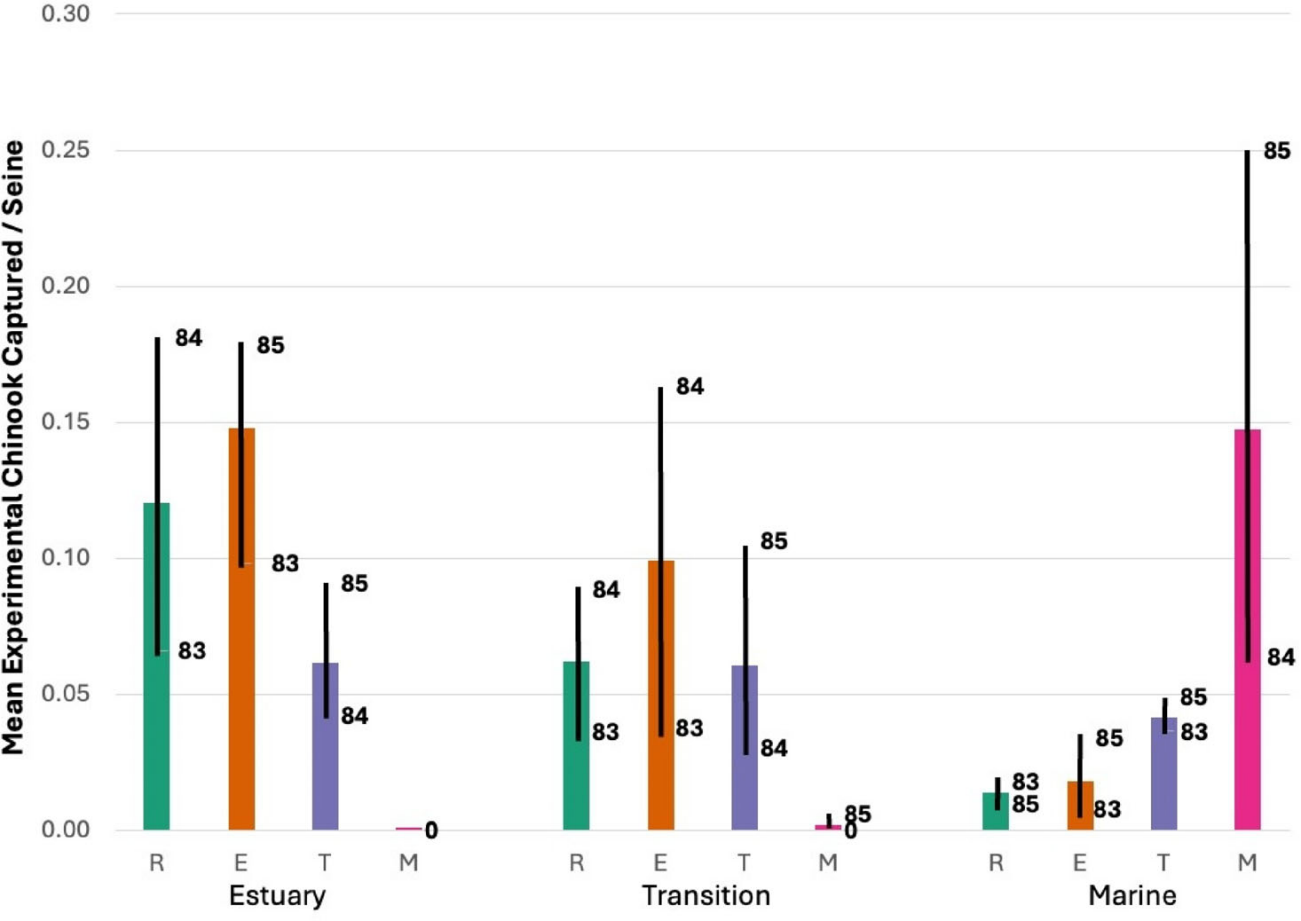
Bar graphs represent mean number of marked Chinook salmon smolts recaptured at estuary, transition and marine ecosystems, arrayed by the location they were released (release group: R – river, E – estuary, T – transition and M – marine). Catch size is corrected for number of seines (CPUE). Vertical bars are range of catch by year from 1983 to 1985. The data depicted in this figure were analysed with catch size on successive trips as a covariate (ANCOVA after Macdonald et al. 1988 – Table 5, with permission) to estimate residency timing each year among the four release groups.

Our initial interpretations from beach seine recoveries of juveniles made each year in the months following releases in the estuary, transition and marine ecosystems (*n* = 747) provided residency estimates of 34 to 47 days in the estuary for the fish that were released there or migrated from the river and 21–23 days for fish released outside the estuary in the transition or marine ecosystem (Table 4, after Macdonald et al., 1988). Residency rate patterns were reported to be consistent among the 3 years but were significantly different among release groups [*p* < 0.05, analysis of covariance (ANCOVA); Table 5, after Macdonald et al., 1988]. A graphical reinterpretation of these data confirmed the original observation of a protracted estuarine residency of approximately 40 days for the bulk of the fish released to freshwater and the estuary (i.e., days between first and last captures annually for 80% recovery of each release group in each ecosystem) or > 70 days for the final capture (i.e., days for 100% recovery) (Figure 4). River‐released fish were previously reported to display longer residence in the entire experimental area (Table 4, after Macdonald et al., 1988) and are now reported as extending their residency in each release location beyond that displayed by fish released in the estuary, transition and marine (Figure 4). Reinterpretation also revealed a tendency for many transition‐released fish to move unexpectedly into the estuary and delay seaward migration for periods similar to those fish released in the estuary and river (Figures 4 and 5). This behaviour hampered dispersal pattern calculations and comparisons in Macdonald et al. (1988). Furthermore, the transition zone was occupied for a considerably longer period of time than previously reported (up to 80 days, Figure 4), particularly in 1984 and 1985. With re‐examination, recoveries of release groups demonstrated a tendency towards fewer recaptures in 1983, particularly among river and estuarine releases in the estuary and the transition ecosystems (Figure 5) and fewer salmon of any species (Table 1). Annual effects were considered previously as being insignificant (Macdonald et al., 1988; Table 5).

**TABLE 4:** Estimates of dispersion rates from the Campbell River estuary – Discovery Passage area of each release group of fish (after Macdonald et al., 1988, with permission).

Release group	Rate	Mean residency time (d)
River	−0.070	47.3
Estuary	−0.010	33.8
Transition	−0.002	23.2
Marine	−0.160	20.7

*Note*: Rates are the slope coefficients that result from the regression model presented in Table 5 and provide estimates of the mean residency time (d) in the Campbell River‐Discovery Passage region. They are calculated from the dispersal rate regression equations, except for transition‐released fish, where the mean number of days between release and capture was used as an estimate of residency time.

**TABLE 5:** Analysis of covariance (ANCOVA) table showing the results of changes in mean catch size among release groups and years with trip.

Source	*df*	SS	F	Probability
Year	2	1.10	0.61	0.54
Release group	3	8.65	3.19	0.02
Year × release group	6	8.80	1.62	0.14
Trip (covariate)	1	7.94	8.80	0.00

*Note*: Analyses were performed using the SAS statistical package (SAS Institute Inc., 1985) sequential sum of squares approach, General Linear Model (GLM) procedure (after Macdonald et al., 1988, with permission).

Using 100% of the catch data, and thus accounting for locations with a small number of recaptures, we expand on the residency findings of Macdonald et al. (1988, Table 4 – 20.7 days for marine releases) to conclude that all release groups moved through the marine ecosystem at nearly twice the rate relative to the other ecosystems (25–37 days vs. 62–85 days, Figure 4 – left hand bars). However, the greater opportunity for the fish to inhabit the deeper locations more common outside the estuary, beneath the reach of a beach seine, and the less confined and larger area represented by the marine location (Table 1), likely influences seining efficiency. Regardless, all release groups were represented in the marine ecosystem recoveries, albeit heavily weighted towards marine releases, which were only rarely caught at other ecosystem locations (Figure 5).

## 4 DISCUSSION

### 4.1 Positive estuary effects on survival were robust

With this final synthesis of data from all adult recoveries, the survival patterns we previously reported remain unchanged: estuary experience by smolts improves adult success. This pattern was detected with a sample size of 163 fish from the 1983 and 1984 releases (Macdonald et al., 1988), and later confirmed following the return of 638 fish from all three release years (Levings et al., 1989). It is now substantiated with the final 1903 recoveries that include fish that extended their time in the ocean. Despite a comparatively good survival of fish released to the marine ecosystem in 1985, smolts that experienced a natural progression through river and estuary ecosystems had consistently strong recoveries as adults in all years, compared to those released to the transition and marine ecosystems. Across years, smolt‐to‐adult survival was 0.34% for fish released in the marine and transition ecosystems and 0.60% for those released in the river and estuary.

Since the middle of the last century, a literature base focused on fine‐scale, habitat specific designs has inferred an estuarine/juvenile salmon survival to adult linkage to explain a decline in populations (Roni et al., 2019; Simenstad et al., 2000), but an unequivocal link has been elusive. The importance of specific habitats is not entirely a function of juvenile Chinook salmon residency; unfrequented habitat is often a source of food, water quality, etc. (Larimore & Garrels, 1985). However, they do spend more time in *ecosystems* with biophysical aspects that promote survival (Levings, 2016). Among the variety of their life‐history strategies, the ocean‐type Chinook salmon juveniles, common to our coastal watersheds, utilize estuary ecosystems for several months (Arbeider et al., 2024). This observation was confirmed with 3 years of effort, tracking juveniles across four Campbell River ecosystems and, combined with our adult recovery patterns, promotes the importance of estuaries during the early Chinook salmon life history. However, basing juvenile and adult survival on a single ecosystem underestimates the role of complex interactions among many variables in several ecosystems; this is considered later in the Discussion.

### 4.2 Juvenile residency estimates support the adult return conclusions

The mean estuarine residency coastwide (Alaska to California) for wild Chinook salmon smolt and fry, estimated at 26 days (range 0–60 days) (Arbeider et al., 2024), is considerably less than the 34–37 day estimates for estuarine and 46–47 day estimates for river‐released fish in this study, and much longer for the final smolts to leave. Together these estimates imply an evolutionary value to a natural progression and extended residency in estuary habitats while moving seaward. However, the early marine ecosystem residence of our juvenile ocean‐type Chinook salmon is less well understood and believed to involve the occupation of deeper water, further from shore where beach seines are ineffective and perhaps created a negative bias in our 25‐day residency estimate. There is a general sense that Chinook smolts lose their affinity for shallow water as they grow (Kjelson et al., 1982; Macdonald et al., 1987) and begin a seaward migration, which may explain a reduced (perhaps by half) estimate of residency near the Discovery Passage beaches in marine ecosystems. Our marine ecosystem residency analysis confirms this observation but identifies milling behaviour between the estuary and transition ecosystems that led to residency estimates for fish released to the transition ecosystem from our reanalysis that were similar among all fish released in the freshwater and estuary ecosystems. Further proof we propose that estuarine conditions provide an attractive opportunity for juvenile salmonids during their migration from a watershed. Milling among large salinity gradients may have implications for osmotic adaptation (Macdonald et al., 1987) or migratory orientation (Healy et al., 2017; McInerney, 1964). In contrast to the transition‐released fish, it is likely that those released in the marine ecosystem could not sense their natal freshwater as an alternative and began moving seaward following release (generally northward; Supporting Information File B). Regardless, if true, there was no evidence that this behaviour created an imprinting impediment among the marine releases, as they returned to their natal habitat in numbers equal or larger than expected from our comparison of catch and escapement.

### 4.3 Seeking environmental factors that may limit survival

Annual differences in survival rates among the fish released to the marine ecosystem provided a significant source of interaction among our main effects (and tab. 2 in Levings et al., 1989) and demonstrates that even a large‐scale focus on the influence of an entire estuary and nearby marine ecosystems by juvenile fish is only one component affecting the survival of an anadromous fish with a complex life history. Our conclusion, and those of numerous other researchers (see Levings, 2016, p. 253), is that salmonids do depend on estuaries, but possible limitations to any anadromous population must also consider watersheds and oceans. In estuaries, there is a consensus that salmonid survival is associated with evolutionary responses to three interacting factors: reduced predation, feeding opportunities, or osmotic stressors (Thorpe, 1994). With consideration to these three factors, our evaluation of the annual variability of environmental characteristics within all of the release ecosystems (Table 1) did not support a relative improvement in marine return success in 1985 but in fact might support the opposite. For example, water released from the Campbell River reservoir in 1985 was 50% of releases in 1983–1984, perhaps resulting in a more stable and predictable salt wedge characteristics and, importantly, diverse estuarine habitat (Macdonald et al., 1987). This may, in turn, directly or indirectly influence salmonid feeding, predation, osmotic adaptation and/or residency of our releases in ecosystem of varying salinity, but less so for fish released outside the river's influence that were recovered in large numbers. Furthermore, perhaps the influence of annual river discharge does extend to the marine areas (e.g., the transition zone), but decreased flows, and associated temperature impacts, have been linked to reduced not increased survival in rivers among other juvenile Chinook salmon populations (Kjelson & Brandes, 1989), and has led to minimum river flow regulations (e.g., Nechako Fisheries Conservation Program, 2005). Nevertheless, physical habitat parameters such as mean temperatures in each ecosystem varied little among years, providing little opportunity with this study to speculate on their influence (Table 1).

Hatchery production from Georgia Strait hatcheries (including the Quinsam River hatchery) increased through the experimental period (Sheng, 2010; Table 1), perhaps leading to reduced feeding opportunities in the estuary. In rivers, managing food webs has been identified as fundamental to ecosystem restoration for target species such as salmonids in the face of uncertain habitat carrying capacity and several pressures among environmental variables often associated with climate change (Naiman et al., 2012). Addressing the potential for competition for food, space and protection was a component to the overarching Campbell River programme. Reclamation efforts by creating vegetated islands in the estuary were designed to offset the demands of hatchery production through the gradual improvement to the food web (Supporting Information File D; Levings & Macdonald, 1991). After 13 years of monitoring, most of the islands and plants remained but large annual variation in sedge survival (Dawe et al., 2000) complicated any inference of an effect at the higher levels of the food web. Unfortunately, a comprehensive Campbell River estuary monitoring programme is not in place now to track long‐term trophic changes, although efforts continue to ensure the long‐term viability of the sedge transplants (Anon, 2020).

Potential hatchery‐reared versus wild salmon interactions were a relatively recent phenomenon in the Campbell River estuary during this experiment because hatchery releases began less than a decade earlier. However, from many perspectives, hatchery interactions in all ecosystems are generally thought to be consequential (Levings et al., 1986; Naiman et al., 2012). For instance, a survey by Weber and Fausch (2003) proposes that large hatchery releases can focus predation pressure on the resulting fish concentrations. Specific to our study, avian predation pressure – though most carefully observed only in 1983 – may have been elevated by our releases only in the marine environment (Macdonald et al., 1987; Macdonald et al., 1988).

**FIGURE 6:**
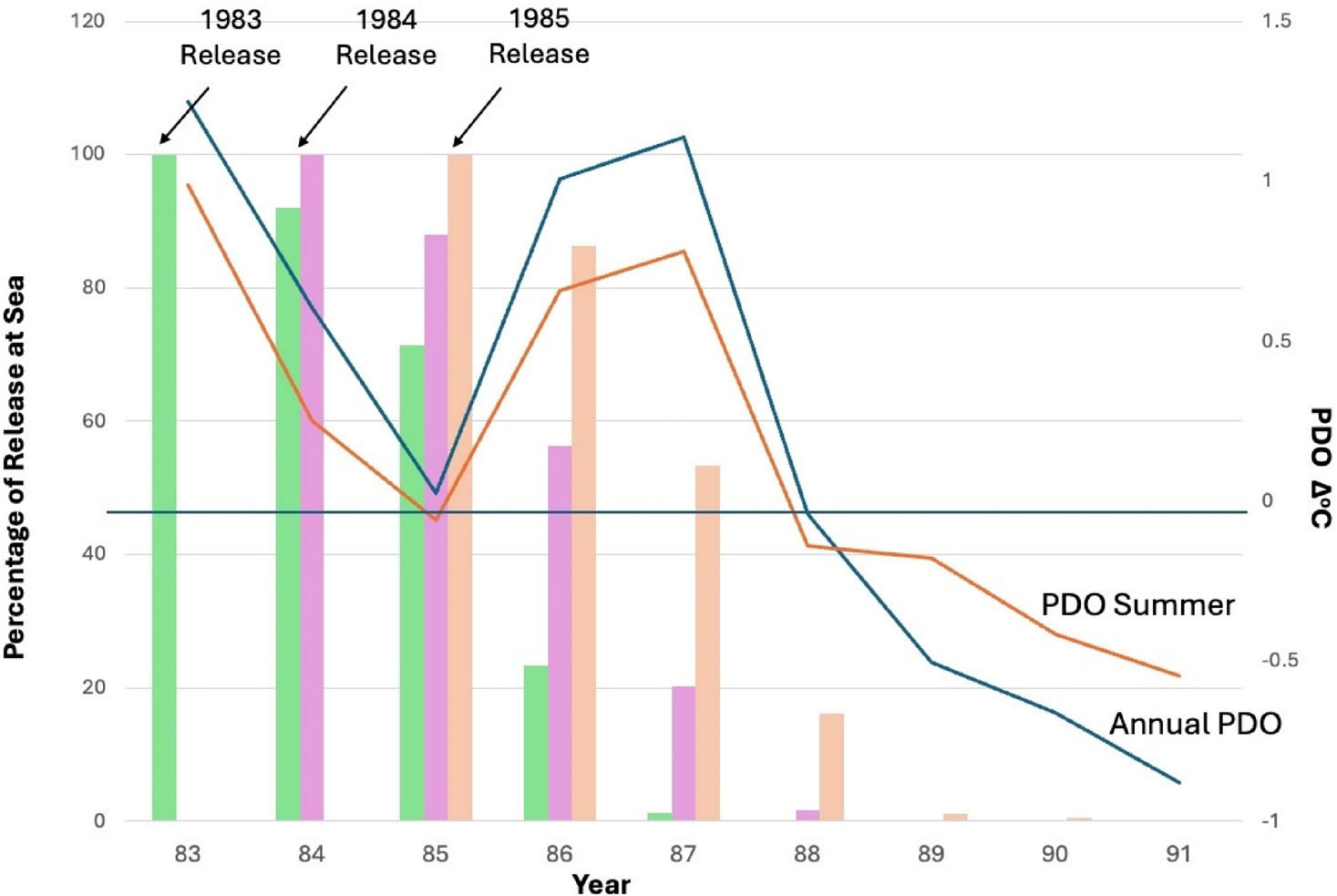
The strength of the annual and summer Pacific Decadal Oscillation (PDO) described as temperature deviations from zero. The PDOs are a surrogate for the conditions in the ocean during those years that experimental fish were present from 1983 to 1991 (annual and summer lines). The three release years appear as vertical bars (1983–1985) and represent the declining portion of each release that remained alive at sea based on the age structure of the returns, as described in the text.

The relative inconsistency in the annual recovery of adults from smolts released in the marine ecosystem may implicate conditions and factors at a larger spatial and temporal scale in ecosystems beyond the experimental area. For example, in 1984 and 1985, Chinook adults were exposed to a larger sport fishing effort in the Strait of Georgia, which subsequently declined through 1990 (English et al., 2002), when the bulk of the 1985 release was returning to spawn. During the study period juvenile salmon migrated to the sea through coastal regions north of Discovery Passage in increasing abundance, notably more pink salmon from the Fraser River and other systems (Table 1). Furthermore, broad geographical Chinook salmon population declines have been linked to sea surface temperatures (SST) and related environmental factors (Dorner et al., 2018), frequently described as Pacific Decadal Oscillations (PDO; Mantua et al., 1997). The mid‐Pacific PDO will have influenced coastal climates at a finer geographical scale, including river discharge, coastal salinity and coastal temperatures (Harrison et al., 2005; Hunt et al., 2024). The SST for the period from the release of the experimental fish in April to the end of each year declined but was on average positive in 1983 and 1984, but near negative in 1985 (National Oceanic and Atmospheric Administration, 2025). In subsequent years, the 1985 release may have been exposed to a more variable environment with both rising and falling temperatures that were generally cooler (Figure 6). However, interpretation may be challenging because salmonid population responses to ocean‐scale climate processes are not necessarily homogeneous among runs even from adjacent watersheds (Levin, 2003). Therefore, regional environmental factors may still have a greater biological influence than ocean‐scale processes (Ohlberger et al., 2016), but perhaps fish released in the first 2 years experienced quite different marine conditions than those released in 1985. Broad‐scale events, such as variable fishing methods in various locations and fluctuations in ocean environments (Supporting Information File A – Table 1), need to be considered as well, and they may be implicated in the inconsistent temporal pattern of age frequency recoveries in this experiment. The apparent randomness in age frequency responses among years and release groups could be attributed to this type of annual variability, but there is little evidence to link age at recovery with a juvenile life history in the 3 years of this experiment. As pointed out by Gosselin et al. (2025), growth rate and length, migration timing and environmental conditions during downstream migration are drivers of age at maturity. In this regard, to our knowledge, estuary conditions have not been considered in this complex of factors.

### 4.4 Future estuary monitoring and experimental design considerations

A case study such as ours complements other large‐scale designs but, for practical reasons, is based on a single Chinook salmon stock within a single watershed. Although clearly scientifically desirable, true spatial (additional watersheds/stocks) and temporal (additional years) replication would significantly increase expenses (sensu Hurlbert, 1984). Here, a study's contribution to a meta‐analysis combined with other case studies in independent locations can be useful (Cottenie & De Meester, 2003). Furthermore, efforts to create replication with multiple release batches were hampered by questions of tag code independence, which is difficult to avoid when raising hatchery fish. Nevertheless, to improve future large‐scale transfer experiments, our foremost recommendation is to consider conducting releases over additional years to account for a greater range in local and oceanic ecosystem conditions, all possibly more observable on a decadal rather than an annual scale. Fundamentally, our experience with this experiment accentuates the importance of considering temporal relative to spatial variation in future study designs. Extrapolation among stocks is likely to be made more reliably than temporal predictions in the midst of climate change. Comparable juvenile salmon habitat exclusion experiments of a 6‐year duration in the Sacramento River estuary (Kjelson & Brandes, 1989), 7 years in the Columbia River estuary (Dietrich et al., 2016) and 12 years in Norway (Gunnerød et al., 1988; Heggberget et al., 1991) provided opportunities to demonstrate the influence of longer‐term temporal factors, such as elevated temperatures and the influence of habitat exclusion, on smolt survival. Lengthy commitments to labour‐intensive programmes to examine factors that limit salmon production are difficult to achieve, but other experimental approaches exist.

In recent years, fisheries research has benefited greatly from extensive long‐term examination of ocean conditions (e.g., Pacific Decadal Oscillation, Mantua et al., 1997; sea level rise and climatic change, Vadeboncoeur, 2016) and river flows (e.g., Morrison & Foreman, 2005; Water Survey of Canada, 2023). Persistent monitoring programmes have provided long‐term and unexpected advantages for the management of salmonid life‐cycle periods, including rearing (Tschaplinski et al., 2025), early marine (Ohlberger et al., 2016), ocean adults (Hinch & Martins, 2011), spawning migration (Macdonald et al., 2010) and spawning habitat (Chhor, 2024). This long‐term research commitment has contributed to the development of regulations for virtually every sector of the Canadian economy. Comparable extended research in estuaries is rare, yet with a mere 3 years of releases from the Campbell watershed, this experiment implies a role for estuaries that is both critical to Chinook conservation and dependent on the annual qualities of other facets of their life history. Limitations responsible for Chinook salmon survival likely vary annually, thereby highlighting an awareness of a synergy only available from long‐term observations to measure many key features of their entire life history.

Large, spatially expansive synoptic designs have also linked the loss of coastal wetland areas to declines in anadromous populations, perhaps with a different degree of inference compared to transfer experiments (Brophy et al., 2019; David et al., 2016; Kehoe et al., 2021; Levings & Thom, 1994). As well, studies comparing smolt‐to‐adult survival in multiple estuaries with varying degree of industrial development (Magnusson & Hilborn, 2003) or chemical contamination (Meador, 2014) using larger hatchery Chinook salmon than ours demonstrated that pristine estuaries produced better smolt‐to‐adult survival than those with more human influence. When combined, the many types of large‐scale experimental approaches and the plethora of small‐scale site‐specific designs in the literature (Roni et al., 2019; Simenstad et al., 2000) contribute to the weight of evidence supporting freshwater and estuarine conservation efforts across the west coast of North America.

### 4.5 Concluding remarks: The regulation and management of estuaries

Chinook salmon juveniles reside *within* the estuarine ecosystem while moving *between* habitat mosaics and emigrate to marine ecosystem seaward in response to complex and interacting factors. At a finer scale, our residency estimates and ecological studies have confirmed that our experimental fish use many mosaics, including sloughs, marshes, beaches and off‐channel habitat in the estuary, as well as kelp and cobble beach habitats in the transition and marine ecosystems (Brown et al., 1987; Supporting Information File F – Table 1). Enhanced survival has been attributed to connected habitat features within the mosaic, including off‐channel marshes (Levings & Macdonald, 1991), tidal influence on water depth (Levy & Northcote, 1982), vegetation (Levy & Northcote, 1981; Sharpe et al., 2019), turbidity (Gregory & Levings, 1996; Sharpe et al., 2019) and flow regimes and water property fluctuations (e.g., salt wedge dynamics; Macdonald et al., 1987). Biological factors such as competing salmonid densities (Levings et al., 1986) or invasive species may also be important (e.g., *Carassius auratus*) (Anon, 2024). Contemporary technologies, such as radio and PIT tags (Hinch et al., 2025 review), show promise to link these mosaics by tracking fine‐scale movements and residency of juvenile salmon between and within the sloughs, marshes, eelgrass beds, gravel beaches and off‐channel habitats that typify estuaries.

As exemplified by some of the seminal papers on the topic (e.g., Hood et al., 2022; Simenstad et al., 2000), an expansion of the fine‐scale site‐specific perspective to the landscape scale can better guide estuarine management. Our case history can help research planners and managers in this regard. For example, marsh reclamation, dike breaching, flow control and other conservation efforts, each argued individually as salmon‐influencing and habitat‐restoring elements of importance, are parts of a system and require consideration at a larger complex landscape perspective. The fine‐scale perspective often aligns with the scale set by development pressures and reclamation opportunities. Thus, regulatory terminology such as ‘critical habitat’, ‘habitat banking’ and ‘indicator species’ have become accepted in our profession, as we seek a path to ‘sustainable development’. But ‘ecological‐based decision making’ has also entered our lexicon and with it a recognition that managing complex environments requires acceptance of perspectives at several scales. A landscape‐level perspective, at a scale corresponding to the ecosystems being examined, can support the conservation of salmonids by promoting the integrity of the entire estuarine ecosystem that is greater than the sum of its parts. This estuary itself is but one of many landscape components responsible for their welfare through their entire life cycle.

## AUTHOR CONTRIBUTIONS

Ideas: Colin D. Levings and J. Steve Macdonald; data generation: Colin D. Levings and J. Steve Macdonald; data analysis: J. Steve Macdonald; manuscript preparation – Colin D. Levings and J. Steve Macdonald.

## Supporting information


**Supporting Information File A:** Information describing juvenile and adult recovery methods, equipment and fishery locations.

**Supporting Information File B:** Background on marine rearing areas of Chinook salmon from Campbell River.

**Supporting Information File C:** Table 1. Number of juvenile Chinook salmon marked with three unique replicate tag codes (N) to identify release location each year, and the number of adults recovered from the fisheries and spawning grounds (C). The ratio of numbers recovered to those released (C/N), with means and standard errors of the mean provides an estimate of survival. Overall survival across all treatments was 0.47%.

**Supporting Information File D:** Photo 1. View of helicopter about to release experimental Chinook salmon smolts at Deepwater Bay, the marine ecosystem site (1983).
Photo 2. Habitat development in the Campbell River estuary. a) artificial island/peninsula constructed with dredged gravel, before sedge transplant in 1983 b) portion of same island in 1985.

**Supporting Information File E:** Table 1: Age distribution of recoveries from releases to each of the four ecosystems each year. Fish were recovered in the fishery and from escapements to the natural spawning grounds and the hatchery from 1983 to 1990. These data are the basis for Figure 3 in the manuscript and Supporting Information Figure 1.
Figure 1: Age frequency of recoveries by release location each year. Release location had an influence on age of recovery: river and estuary releases were older in 1983 and younger in 1984, whereas marine release recoveries were primarily 4 years old (χ^2^, *p* < 0.05; Figure 3 – manuscript). Age and release location were not associated in 1985 (*p* > 0.05).

**Supporting Information File F:** Table 1. Number of sample sites characterized by various habitat types across the three ecosystems where released marked smolts were recaptured in the residency estimate study. Not all estuary sites are shown in Figure 1 because some sites overlapped or fall outside the image at the scale shown. From Brown et al. (1987).

## Data Availability

Some data for this project are provided electronically in Supporting Information Files A–F. Additional raw data from this project are available on Government of Canada Federal Science Library website (https://science-libraries.canada.ca/eng/home/) as Data Reports and can be located by searching the site on the authors' names.

## References

[jfb70443-bib-0001] Anon . (2020). Passing the baton of environmental stewardship to seven generations on a Vancouver Island estuary. Campbell River Mirror, October 20, 2020. https://www.campbellrivermittor.com/news/passing-the-baton-of-environmental-stwardship-to-seven-generations-on-a-vancouver-island-estuary/

[jfb70443-bib-0002] Anon . (2023). Coastwide Evaluation and Classification of Pacific Region Estuaries based on Anthropogenic Activities and Significant Fish Habitat. Science Advisory Report 2023/030. https://www.dfo-mpo.gc.ca/csas-sccs/Publications/SAR-AS/2023/2023_039-eng.html

[jfb70443-bib-0003] Anon . (2024). Fishy situation: Over 1,700 goldfish pulled from Campbell River Pond. Campbell River Mirror. October 22, 2024. https://www.campbellrivermirror.com/writers/robin.grant

[jfb70443-bib-0004] Arbeider, M. , Pemberton‐Renaud, V. , Hodgson, E. E. , & Moore, J. W. (2024). The estuarine growth and residency of juvenile Pacific salmon in North America: A compilation of empirical data. Canadian Journal of Fisheries and Aquatic Sciences, 81, 253–267. 10.1139/cjfas-2023-0225

[jfb70443-bib-0005] Atlas, W. I. , Ban, N. C. , Moore, J. W. , Tuohy, A. M. , Greening, S. , Reid, A. J. , Morven, N. , White, E. , Housty, W. G. , Housty, J. A. , Service, C. N. , Greba, L. , Harrison, S. , Sharpe, C. , Butts, K. I. R. , Shepert, W. M. , Sweeney‐Bergen, E. , Macintyre, D. , Sloat, M. R. , & Connors, K. (2021). Indigenous systems of management for culturally and ecologically resilient Pacific salmon (*Oncorhynchus* spp.) fisheries. Bioscience, 71(2), 186–204. 10.1093/biosci/biaa144 33613129 PMC7882363

[jfb70443-bib-0006] Bell, L. M. , & Thompson, J. M. (1977). The Campbell River estuary: status of environmental knowledge to 1977. Report of the Estuary Working Group, Department of Fisheries and the Environment, Regional Board Pacific Region. 398p.

[jfb70443-bib-0007] Blackmun, G. J. , Lukyn, B. V. , McLean, W. E. , & Ewart, D. (1985). Quinsam watershed study: 1983. Canadian Manuscript Report of Fisheries and Aquatic Sciences, 1832. 65p.

[jfb70443-bib-0008] Brophy, L. S. , Greene, C. M. , Hare, V. C. , Holycross, B. , Lanier, A. , Heady, W. N. , & Dana, R. (2019). Insights into estuary habitat loss in the western United States using a new method for mapping maximum extent of tidal wetlands. PLoS One, 14(8), e0218558. 10.1371/journal.pone.0218558 31412030 PMC6693690

[jfb70443-bib-0009] Brown, T. J. , McAllister, C. D. , & Kotyk, M. S. (1987). A summary of the salmonid catch‐data from Campbell River estuary and Discovery Passage for the years 1982‐1986. Can. Data Rep. Fish. Aquat. Sci. 650. 99p.

[jfb70443-bib-0010] Bugnot, A. B. , Gorman, D. , Steven, A. D. L. , & Vanderklift, M. A. (2024). Measuring fisheries outcomes from coastal wetland restoration: A review and meta‐analysis. Restoration Ecology, 33(1). 10.1111/rec.14324

[jfb70443-bib-0012] Chalifour, L. , Scott, D. C. , MacDuffee, M. , Stark, S. , Dower, J. F. , Beacham, T. D. , & Baum, J. K. (2021). Chinook salmon exhibit long‐term rearing and early marine growth in the Fraser River, British Columbia, a large urban estuary. Canadian Journal of Fisheries and Aquatic Sciences, 78(5), 539–550. 10.1139/cjfas-2020-0247

[jfb70443-bib-0013] Chhor, A. (2024). Drought resilience for Nicola River salmon: Eight policy solutions to benefit wild salmon in an age of water scarcity. Raincoast Conservation Foundation: 16p. 10.70766/912.482

[jfb70443-bib-0014] Clarke, W. C. , & Blackburn, J. (1977). A seawater challenge test to measure smolting of juvenile salmon. Fish. Mar. Serv. Res. Dev. Tech. Rep. 705: 11p.

[jfb70443-bib-0015] Clarke, W. C. , & Shelbourn, J. E. (1985). Growth and development of seawater adaptability by juvenile fall chinook salmon (*Oncorhynchus tshawytscha*) in relation to temperature. Aquaculture, 45(1–4), 21–31. 10.1016/0044-8486(85)90255-8

[jfb70443-bib-0016] Cottenie, K. , & De Meester, L. (2003). Comment to Oksanen (2001): Reconciling Oksanen (2001) and Hurlbert (1984). Oikos, 100(2), 394–396. Portico. 10.1034/j.1600-0706.2003.11953.x

[jfb70443-bib-0017] David, A. T. , Simenstad, C. A. , Cordell, J. R. , Toft, J. D. , Ellings, C. S. , Gray, A. , & Berge, H. B. (2016). Wetland loss, juvenile salmon foraging performance, and density dependence in Pacific northwest estuaries. Estuaries and Coasts, 39, 767–780. 10.1007/s12237-015-0041-5

[jfb70443-bib-0018] Davies, G. M. , & Gray, A. (2015). Don't let spurious accusations of pseudoreplication limit our ability to learn from natural experiments (and other messy kinds of ecological monitoring). Ecology and Evolution, 5(22), 5295–5304. 10.1002/ece3.1782 30151132 PMC6102510

[jfb70443-bib-0019] Davis, M. J. , Woo, I. , De La Cruz, S. E. , Ellings, C. S. , Hodgson, S. , & Nakai, G. (2024). Allochthonous marsh subsidies enhances food web productivity in an estuary and its surrounding ecosystem mosaic. PLoS One, 19(2), e0296836. e0331309. 10.1371/journal.pone.0331309 38421974 PMC10903911

[jfb70443-bib-0020] Dawe, N. K. , Bradfield, G. E. , Boyd, W. S. , Trethewey, D. E. , & Zolbrod, A. N. (2000). Marsh creation in a northern Pacific estuary: Is thirteen years of monitoring vegetation dynamics enough? Conservation Ecology, 4(2), 12. 10.1371/journal.pone.0296836

[jfb70443-bib-0021] Des Roches, S. , Accola, K. L. , Faulkner, H. S. , Morgan, J. R. , Perla, B. S. , Metler, M. , Dethier, M. N. , & Toft, J. D. (2024). Shoreline restoration including armor removal and log placement affect ecosystem recovery through time. Restoration Ecology, 32(4), e14097. 10.1111/rec.14097

[jfb70443-bib-0022] Diefenderfer, H. L. , Steyer, G. D. , Harwell, M. C. , LoSchiavo, A. J. , Neckles, H. A. , Burdick, D. M. , Johnson, G. E. , Buenau, K. E. , Trujillo, E. , Callaway, J. C. , Thom, R. M. , Ganju, N. K. , & Twilley, R. R. (2021). Applying cumulative effects to strategically advance large‐scale ecosystem restoration. Frontiers in Ecology and the Environment, 19(2), 108–117. 10.1002/fee.2274 PMC8597595 34795552

[jfb70443-bib-0023] Dietrich, J. , Eder, K. , Thompson, D. , Buchanan, R. , Skalski, J. , McMichael, G. , Fryer, D. , & Loge, F. (2016). Survival and transit of in‐river and transported yearling Chinook salmon in the lower Columbia River and estuary. Fisheries Research, 183, 435–446. 10.1016/j.fishres.2016.07.005

[jfb70443-bib-0024] Dorner, B. , Catalano, M. J. , & Peterman, R. M. (2018). Spatial and temporal patterns of covariation in productivity of Chinook salmon populations of the northeastern Pacific Ocean. Canadian Journal of Fisheries and Aquatic Sciences, 75(7), 1082–1095. 10.1139/cjfas-2017-0197

[jfb70443-bib-0025] Egan, V. G. (1988). Tyee: The story of the Tyee Club of British Columbia (p. 208). Ptarmigan Press.

[jfb70443-bib-0026] Elliott, J. M. (1977). Some methods for the statistical analysis of samples of benthic invertebrates. Freshwater Biological Association Scientific Publication, 25, 1–160.

[jfb70443-bib-0027] English, K. , Searing, G. P. , & Nagtegaal, D. A. (2002). Review of the strait of Georgia recreational creel survey, 1983‐1999. Canadian Technical Report of Fisheries and Aquatic Sciences, 2414. 81p.

[jfb70443-bib-0028] Goodman, D. , & Vroom, P. R. (1974). An assessment of the impact of proposed marina development on the fisheries resources of the Campbell River estuary. Technical Report Series No. PAC/T‐74‐13.Vancouver: Fisheries and Marine Service, Southern Operations Branch.

[jfb70443-bib-0029] Gosselin, J. L. , Sandford, B. P. , O'Brien, C. S. , & Buhle, E. R. (2025). Biological, freshwater, and marine drivers of age at maturity in wild Chinook Salmon. bioRxiv, 2025–12. 10.64898/2025.12.13.694134

[jfb70443-bib-0030] Gregory, R. S. , & Levings, C. D. (1996). The effects of turbidity and vegetation on the risk of juvenile salmonids, *Oncorhynchus spp*., to predation by adult cutthroat trout, *O. clarkii* . Environmental Biology of Fishes, 47, 279–288. 10.1007/bf00000500

[jfb70443-bib-0031] Gunnerød, T. B. , Hvidsten, N. A. , & Heggberget, T. G. (1988). Open sea releases of Atlantic salmon smolts, *Salmo salar*, in central Norway, 1973–83. Canadian Journal of Fisheries and Aquatic Sciences, 45(8), 1340–1345. 10.1139/f88-157

[jfb70443-bib-0032] Haig‐Brown, R. (1946). A River Never Sleeps. Collins.

[jfb70443-bib-0033] Harrison, P. J. , Whitney, F. , Mackas, D. , Beamish, R. , Trudel, M. , & Perry, I. (2005). Changes in coastal ecosystems in the NE Pacific Ocean. In Proceedings of the International Symposium on the long‐term variations in the coastal environments and ecosystems, Sept 27–28, Matsuyama, Japan, pp. 17–44. https://repository.hkust.edu.hk/ir/bitstream/1783.1-2345/1/HarrisonJapanMSFinalChangesecosystem.pdf

[jfb70443-bib-0034] Healey, M. C. (1982). Juvenile Pacific salmon in estuaries: The life support system. In V. S. Kennedy (Ed.), Estuarine comparisons, pp. 315‐341. Proceedings of the sixth biennial international estuarine research conference, Gleneden Beach, Oregon, November 1‐6, 1981 (p. 709). Academic Press. 10.1016/b978-0-12-404070-0.50025-9

[jfb70443-bib-0035] Healey, M. C. (1983). Coastwide distribution and ocean migration patterns of Stream‐and Ocean‐type Chinook Salmon, *Oncorhynchus tshawytscha* . The Canadian Field‐Naturalist, 97(4), 427–433. http://www.biodiversitylibrary.org/item/89042

[jfb70443-bib-0036] Healy, S. J. , Hinch, S. G. , Porter, A. D. , Rechisky, E. L. , Welch, D. W. , Eliason, E. J. , Lotto, A. G. , & Furey, N. B. (2017). Route‐specific movements and survival during early marine migration of hatchery steelhead *Oncorhynchus mykiss* smolts in coastal British Columbia. Marine Ecology Progress Series, 577, 131–147. 10.3354/meps12238

[jfb70443-bib-0037] Hecht, B. C. , Matala, A. P. , Hess, J. E. , & Narum, S. R. (2015). Environmental adaptation in Chinook salmon (*Oncorhynchus tshawytscha*) throughout their north American range. Molecular Ecology, 24(22), 5573–5595. 10.1111/mec.13409 26465117

[jfb70443-bib-0038] Heggberget, T. G. , Hvidsten, N. A. , Gunnerød, T. B. , & Møkkelgjerd, P. I. (1991). Distribution of adult recaptures from hatchery‐reared Atlantic salmon (*Salmo salar*) smolts released in and off‐shore of the river Surna, western Norway. Aquaculture, 98(1–3), 89–96. 10.1016/0044-8486(91)90374-G

[jfb70443-bib-0096] Hinch, S. G. , Cooke, S. J. , & Young, N. (2025). Once considered a “disruptive science”, biotelemetry is now among the most trusted and relevant approaches informing salmon fisheries management. Canadian Journal of Fisheries and Aquatic Sciences, 82, 1–16.

[jfb70443-bib-0039] Hinch, S. G. , & Martins, E. G. (2011). Climate Change. Technical Report 9, *In*: Cohen, B.I. 2012. The uncertain future of Fraser River sockeye. Vol 2 Causes of the decline. 236p. https://publications.gc.ca/collections/collection_2012/bcp-pco/CP32-93-2012-2-eng.pdf

[jfb70443-bib-0040] Hood, W. G. , Blauvelt, K. , Bottom, D. L. , Castro, J. M. , Johnson, G. E. , Jones, K. K. , Krueger, K. L. , Thom, R. M. , & Wilson, A. (2022). Using landscape ecology principles to prioritize habitat restoration projects across the Columbia River estuary. Restoration Ecology, 30(3), e13519. 10.1111/rec.13519

[jfb70443-bib-0041] Houde, A. L. S. , Günther, O. P. , Strohm, J. , Ming, T. J. , Li, S. , Kaukinen, K. H. , Patterson, D. A. , Farrell, A. P. , Hinch, S. G. , & Miller, K. M. (2019). Discovery and validation of candidate smoltification gene expression biomarkers across multiple species and ecotypes of Pacific salmonids. Conservation Physiology, 7(1), 66p https://www.dfo-mpo.gc.ca/csas-sccs/Publications/SAR-AS/2023/2023_039-eng.html 10.1093/conphys/coz051 PMC6788492 31620289

[jfb70443-bib-0042] Hunt, B. P. , Alin, S. , Bidlack, A. , Diefenderfer, H. L. , Jackson, J. M. , Kellogg, C. T. , Kiffney, P. , St. Pierre, K. A. , Carmack, E. , Floyd, W. C. , Hood, E. , Horner‐Devine, A. R. , Levings, C. , & Vargas, C. A. (2024). Advancing an integrated understanding of land–ocean connections in shaping the marine ecosystems of coastal temperate rainforest ecoregions. Limnology and Oceanography, 69(12), 3061–3096. 10.1002/lno.12724

[jfb70443-bib-0043] Hurlbert, S. H. (1984). Pseudoreplication and the design of ecological field experiments. Ecological Monographs, 54(2), 121–187. 10.2307/1942661

[jfb70443-bib-0044] Kask, B. A. , Brown, T. J. , & McAllister, C. D. (1986). Nearshore epibenthos of the Campbell River estuary and Discovery passage, 1982, in relation to juvenile chinook diets. Can. Tech. Rep. Fish. Aquat. Sci. 1449p.

[jfb70443-bib-0097] Kask, B. A. , Brown, T. J. , & McAllister, C. D. (1988). Nearshore epibenthos of the Campbell River estuary and Discovery passage, 1983, in relation to juvenile chinook diets. Can. Tech. Rep. Fish. Aquat. Sci. 1616. 71 p.

[jfb70443-bib-0045] Kehoe, L. J. , Lund, J. , Chalifour, L. , Asadian, Y. , Balke, E. , Boyd, S. , Carlson, D. , Casey, J. M. , Connors, B. , Cryer, N. , Drever, M. C. , Hinch, S. , Levings, C. , MacDuffee, M. , McGregor, H. , Richardson, J. , Scott, D. C. , Stewart, D. , Vennesland, R. G. , & Martin, T. G. (2021). Conservation in heavily urbanized biodiverse regions requires urgent management action and attention to governance. Conservation Science and Practice, 3(2), Portico. 10.1111/csp2.310

[jfb70443-bib-0046] Kennedy, L. A. , Juanes, F. , & El‐Sabaawi, R. (2018). Eelgrass as valuable nearshore foraging habitat for juvenile Pacific salmon in the early marine period. Marine and Coastal Fisheries, 10(2), 190–203. 10.1002/mcf2.10018

[jfb70443-bib-0047] Kjelson, M. A. , & Brandes, P. L. (1989). The use of smolt survival estimates to quantify the effects of habitat changes on salmonid stocks in the Sacramento‐San Joaquin rivers, California. In C. D. Levings , L. B. Holtby , & M. A. Henderson (Eds.), Proceedings of the National Workshop on effects of habitat alteration on salmonid stocks (pp. 116–122). Fisheries and Oceans Canada, Communications Directorate, 1989. Can. Spec. Pub. Fish. Aquat. Sci. 105. 199p.

[jfb70443-bib-0048] Kjelson, M. A. , Raquel, P. F. , & Fisher, F. W. (1982). Life history of fall‐run juvenile chinook salmon, *Oncorhynchus tshawytscha*, in the Sacramento‐San Joaquin estuary, California. In V. S. Kennedy (Ed.), Estuarine Comparisons *Proceedings of the Sixth Biennial International Estuarine Research Conference, Gleneden Beach, Oregon, November 1‐6, 1981* (pp. 393–411). Academic Press. 709p. 10.1016/b978-0-12-404070-0.50029-6

[jfb70443-bib-0049] Kotyk, M. S. , Levings, C. D. , Brown, T. J. , McAllister, C. D. , Macdonald, J. S. , Fagerlund, U. H. M. , & McBride, J. R. (1985). An account of an experimental release of marked juvenile chinook to freshwater, estuarine, and marine habitats near Campbell River, B.C. 1984. Can. Tech. Rep. Fish. Aquat. Sci. 1397. 31p.

[jfb70443-bib-0050] Larimore, R. W. , & Garrels, D. D. (1985). Assessing habitats used by warm water stream fishes. Fisheries, 10, 16–18. 10.1577/1548-8446(1985)010<0010:AHUBWS>2.0.CO;2

[jfb70443-bib-0051] Lerner, J. E. , & Hunt, B. P. (2024). Stable isotopes delineate regional pelagic food web structure in British Columbia's coastal ocean. Canadian Journal of Fisheries and Aquatic Sciences, 81(4), 368–386. 10.1139/cjfas-2023-0057

[jfb70443-bib-0052] Levin, P. S. (2003). Regional differences in responses of chinook salmon populations to large‐scale climatic patterns. Journal of Biogeography, 30(5), 711–717. Portico. 10.1046/j.1365-2699.2003.00863.x

[jfb70443-bib-0053] Levings, C. D. (1984). Commentary: Progress in attempts to test the null hypothesis that juvenile salmonids aren't dependent on estuaries. p. 287‐296 *In*: The influence of ocean conditions on the production of salmonids in the North Pacific. W.G. Pearcy, (Ed.). A Workshop Sponsored by the Cooperative Institute for Marine Resources Studies, Nov. 8‐10. 1983, Newport, Oregon.

[jfb70443-bib-0054] Levings, C. D. (2016). Ecology of salmonids in estuaries around the world: Adaptations, habitats, and conservation. UBC Press. 388 pages and on line supplemental material. 10.59962/9780774831758

[jfb70443-bib-0055] Levings, C. D. , Kotyk, M. S. , Brown, T. J. , McAllister, C. D. , Macdonald, J. S. , Fagerlund, U. H. M. , & McBride, J. (1984). An account of an experimental release of marked juvenile Chinook to freshwater, estuarine and marine habitats near Campbell River, B.C. Can. Tech. Rep. Fish. Aquat. Sci. 1269. 35p.

[jfb70443-bib-0056] Levings, C. D. , & Macdonald, J. S. (1991). Rehabilitation of estuarine fish habitat at Campbell River, British Columbia. American Fisheries Society Symposium, 10, 176–190. 10.1201/9781439822678.ch21

[jfb70443-bib-0098] Levings, C.D. , Macdonald, J.S. and L. Lu (in prep).Juvenile salmon ecology at the northern outlets of the Strait of Georgia. 27 p

[jfb70443-bib-0057] Levings, C. D. , McAllister, C. D. , & Chang, B. D. (1986). Differential use of the Campbell River estuary, British Columbia by wild and hatchery‐reared juvenile Chinook salmon (*Oncorhynchus tshawytscha*). Canadian Journal of Fisheries and Aquatic Sciences, 43(7), 1386–1397. 10.1139/f86-172

[jfb70443-bib-0058] Levings, C. D. , McAllister, C. D. , Macdonald, J. S. , Brown, T. J. , Kotyk, M. S. , & Kask, B. A. (1989). Chinook salmon and estuarine habitat: A transfer experiment can help evaluate estuary dependency. p. 116–122. In C. D. Levings , L. B. Holtby , & M. A. Henderson (Eds.), Proceedings of the National Workshop on Effects of Habitat Alteration on Salmonid Stocks. Fisheries and Oceans Canada, Communications Directorate, 1989. *Can. Spec. Pub. Fish. Aquat. Sci* . (Vol. 105, p. 199).

[jfb70443-bib-0059] Levings, C. D. , & Thom, R. M. (1994). Habitat changes in Georgia Basin: implications for resource management and restoration. p. 330–351. *In*: Review of the Marine Environment and Biota of Strait of Georgia, Puget Sound, and Juan de Fuca Strait. Edited by Wilson, R.C.H., Beamish, R.J., Aitkens, F., & J. Bell. Proc. BC/Washington Symposium on the Marine Environment, January 13–14 1994. Can. Tech. Rep. Fish. Aquat. Sci. 1948. 390p.

[jfb70443-bib-0060] Levy, D. A. , & Northcote, T. G. (1981). The distribution and abundance of juvenile salmon in marsh habitats of the Fraser River estuary (p. 117). Westwater Research Centre, University of British Columbia. Technical Report 25.

[jfb70443-bib-0061] Levy, D. A. , & Northcote, T. G. (1982). Juvenile salmon residency in a marsh area of the Fraser River estuary. Canadian Journal of Fisheries and Aquatic Sciences, 39(2), 270–276. 10.1139/f82-038

[jfb70443-bib-0062] Lukyn, B. V. , Ewart, D. , Blackmun, G. J. , & McLean, W. E. (1985). Quinsam watershed study: First addendum (Jan.‐Aug.1984). Canadian Data Report of Fisheries and Aquatic Sciences, 523. vi, 34p.

[jfb70443-bib-0063] Macdonald, J. S. , Birtwell, I. K. , & Kruzynski, G. M. (1987). Food and habitat utilization by juvenile salmonids in the Campbell River estuary. Canadian Journal of Fisheries and Aquatic Sciences, 44(6), 1233–1246. 10.1139/f87-146

[jfb70443-bib-0064] Macdonald, J. S. , Levings, C. D. , McAllister, C. D. , Fagerlund, U. H. M. , & McBride, J. R. (1988). A field experiment to test the importance of estuaries for Chinook salmon (*Oncorhynchus tshawytscha*) survival: Short term results. Canadian Journal of Fisheries and Aquatic Sciences, 45, 1366–1377. 10.1139/f88-160

[jfb70443-bib-0065] Macdonald, J. S. , Patterson, D. A. , Hague, M. J. , & Guthrie, I. C. (2010). Improving the sustainability of sockeye salmon fisheries by modeling the influence of environmental factors on spawning migration success. Transactions of the American Fisheries Society, 139(3), 768–782. 10.1577/T08-223.1

[jfb70443-bib-0066] MacDuffee, M. , Barrett‐Lennard, L. , Chhor, A. , Dennert, A. M. , Ross, P. S. , Scott, D. C. , Vergara, V. , & Walters, K. (2023). Will Canada permit killer whale extinction? Science, 380(6652), 1330. 10.1126/science.adi5984 37384681

[jfb70443-bib-0067] Magnusson, A. , & Hilborn, R. (2003). Estuarine influence on survival rates of coho (*Oncorhynchus kisutch*) and Chinook salmon (*Oncorhynchus tshawytscha*) released from hatcheries on the US Pacific coast. Estuaries, 26, 1094–1103. 10.1007/bf02803366

[jfb70443-bib-0068] Mantua, N. J. , Hare, S. R. , Zhang, Y. , Wallace, J. M. , & Francis, R. C. (1997). A Pacific interdecadal climate oscillation with impacts on salmon production. Bulletin of the American Meteorological Society, 78(6), 1069–1080. 10.1175/1520-0477(1997)078<1069:apicow>2.0.co;2

[jfb70443-bib-0095] McInerney, J. E. (1964). Salinity preference: an orientation mechanism in salmon migration. Journal of the Fisheries Board of Canada, 21(5), 995–1018.

[jfb70443-bib-0069] Meador, J. P. (2014). Do chemically contaminated river estuaries in Puget Sound (Washington, USA) affect the survival rate of hatchery‐reared Chinook salmon? Canadian Journal of Fisheries and Aquatic Sciences, 71(1), 162–180. 10.1139/cjfas-2013-0130

[jfb70443-bib-0070] Millar, R. B. , & Anderson, M. J. (2004). Remedies for pseudoreplication. Fisheries Research, 70(2–3), 397–407. 10.1016/j.fishres.2004.08.016

[jfb70443-bib-0071] Morrison, J. , & Foreman, M. G. (2005). Forecasting Fraser River flows and temperatures during upstream salmon migration. Journal of Environmental Engineering and Science, 4(2), 101–111. 10.1139/s04-046

[jfb70443-bib-0072] Naiman, R. J. , Alldredge, J. R. , Beauchamp, D. A. , Bisson, P. A. , Congleton, J. , Henny, C. J. , Huntly, N. , Lamberson, R. , Levings, C. , Merrill, E. N. , Pearcy, W. G. , Rieman, B. E. , Ruggerone, G. T. , Scarnecchia, D. , Smouse, P. E. , & Wood, C. C. (2012). Developing a broader scientific foundation for river restoration: Columbia River food webs. PNAS, 109(52), 21201–21207. 10.1073/pnas.1213408109 23197837 PMC3535603

[jfb70443-bib-0073] National Oceanic and Atmospheric Administration . (2025). Pacific Decadal Oscillation January 1854‐March 2025. https://www.ncei.noaa.gov/access/monitoring/pdo/

[jfb70443-bib-0074] Nechako Fisheries Conservation Program . (2005). Nechako Fisheries Conservation Program Decision Record 2005‐06‐03. 14p. https://www.nfcp.org

[jfb70443-bib-0075] Ohlberger, J. , Scheuerell, M. D. , & Schindler, D. E. (2016). Population coherence and environmental impacts across spatial scales: A case study of Chinook salmon. Ecosphere, 7(4), e01333. 10.1002/ecs2.1333

[jfb70443-bib-0076] Oksanen, L. (2001). Logic of experiments in ecology: Is pseudoreplication a pseudoissue? Oikos, 94(1), 27–38. 10.1034/j.1600-0706.2001.11311.x

[jfb70443-bib-0077] Quinn, T. P. (2018). The behavior and ecology of Pacific Salmon and Trout (2nd ed., p. 363). University of Washington Press.

[jfb70443-bib-0078] Raymond, B. A. , Wayne, M. M. , & Morrison, J. A. (1985). Vegetation, invertebrate distribution and fish utilization of the Campbell River estuary, British Columbia. Canadian Manuscript Report of Fisheries and Aquatic Sciences, 1829, 46p.

[jfb70443-bib-0079] Reimers, P. E. (1973). The length of residence of juvenile fall Chinook salmon in Sixes River, Oregon. Research Reports of the Fish Commission of Oregon, 4(2), 3–42. https://ir.library.oregonstate.edu/concern/graduate_thesis_or_dissertations/9z903242m

[jfb70443-bib-0080] Roni, P. , Hall, J. E. , Drenner, S. M. , & Arterburn, D. (2019). Monitoring the effectiveness of floodplain habitat restoration: A review of methods and recommendations for future monitoring. Wiley Interdisciplinary Reviews: Water, 6(4), e1355. 10.1002/wat2.1355

[jfb70443-bib-0081] Sharpe, C. , Carr‐Harris, C. , Arbeider, M. , Wilson, S. M. , & Moore, J. W. (2019). Estuary habitat associations for juvenile Pacific salmon and pelagic fish: Implications for coastal planning processes. Aquatic Conservation: Marine and Freshwater Ecosystems, 29(10), 1636–1656. 10.1002/aqc.3142

[jfb70443-bib-0082] Sheng, M. (2010). An Overview of Coho and Chinook Hatchery Facilities in the Strait of Georgia. http://www.stateofthesalmon.org/conference2010/wdownloads/Thurs_presentations/Session_3/Sheng_Georgia%20StraitCoho%20Chinook%20May%204%2010%203.pdf

[jfb70443-bib-0083] Simenstad, C. A. , Hood, W. G. , Thom, R. M. , Levy, D. A. , & Bottom, D. L. (2000). Landscape structure and scale constraints on restoring estuarine wetlands for Pacific coast juvenile fishes. In M. P. Weinstein & D. A. Kreeger (Eds.), Concepts and controversies in tidal marsh ecology (pp. 597–630). Kluwer Academic Publishers. 10.1007/0-306-47534-0_28

[jfb70443-bib-0084] Stewart, D. , Lievesley, M. , Paterson, J. E. , Hennigar, D. , Ingham, R. , Knight, R. , Mason, B. , & Balke, E. (2024). Factors influencing the resilience of created tidal marshes in the Fraser River estuary, British Columbia. Wetlands, 44(5), 44–53. 10.1007/s13157-024-01802-x

[jfb70443-bib-0085] Thomson, R. E. (1981). Oceanography of the British Columbia coast, Canadian special publication of fisheries and aquatic science. Fisheries and Oceans Canada, 56, 291p.

[jfb70443-bib-0086] Thorpe, J. E. (1994). Salmonid fishes and the estuarine environment. Estuaries, 17(1), 76–93. 10.2307/1352336

[jfb70443-bib-0087] Tschaplinski, P. J. , Reid, D. A. , Pike, R. G. , & Spittlehouse, D. L. (2025). Long term forestry and climate change effects on watershed processes and salmonid populations at Carnation Creek, Vancouver Island, British Columbia. Frontiers in Ecology and Evolution, 30, 1–21. 10.3389/fevo.2025.1704400

[jfb70443-bib-0088] Tutty, B. D. , Raymond, B. A. , & Conlin, K. (1983). Estuarine restoration and salmonid utilization of a previously dyked slough in the Englishman River estuary, Vancouver Island, British Columbia. Canadian Manuscript Report of Fisheries and Aquatic Sciences, 1689, 51p.

[jfb70443-bib-0089] Vadeboncoeur, N. (2016). Perspectives on Canada's west coast region. In D. S. Lemmen , F. J. Warren , T. S. James , & C. S. L. Mercer Clarke (Eds.), Canada's marine coasts in a changing climate (pp. 207–225). Government of Canada. https://www.nrcan.gc.ca/sites/www.nrcan.gc.ca/files/earthsciences/files/pdf/NRCAN_fullBook%20%20accessible.pdf. (accessed Dec 29 2023)

[jfb70443-bib-0090] Van Cise, A. M. , Hanson, M. B. , Emmons, C. , Olsen, D. , Matkin, C. O. , Wells, A. H. , & Parsons, K. M. (2024). Spatial and seasonal foraging patterns drive diet differences among north Pacific resident killer whale populations. Royal Society open. Science, 11(9), 1–19. 10.1098/rsos.240445 rsos240445. PMC11409894 39295918

[jfb70443-bib-0091] Water Survey of Canada . (2023). About the Water Survey of Canada. https://www.canada.ca/en/environment-climate-change/services/water-overview/quantity/monitoring/survey/about.html (accessed Dec 29 2023)

[jfb70443-bib-0092] Weber, E. D. , & Fausch, K. D. (2003). Interaction between hatchery and wild salmonids in streams: Differences in biology and evidence of competition. Canadian Journal of Fisheries and Aquatic Sciences, 60, 1018–1036. 10.1139/f03-087

[jfb70443-bib-0093] Welch, D. W. , Porter, A. D. , & Rechisky, E. L. (2021). A synthesis of the coast‐wide decline in survival of west coast Chinook Salmon (*Oncorhynchus tshawytscha*, Salmonidae). Fish and Fisheries, 22(1), 194–211. 10.1111/faf.12514

[jfb70443-bib-0094] Zar, J. H. (1974). Biostatistical analysis (p. 620). Prentice‐Hall Inc..

