# Supplementary material for: Historical case study of a transfer experiment that demonstrated the importance of the estuarine ecosystem for the survival of Chinook salmon (Oncorhynchus tshawytscha) at the Campbell River estuary, British Columbia, Canada
Source: J Fish Biol. 2026 May 20;109(2):1125–40. doi: 10.1111/jfb.70443 (PMC13521722; doi:10.1111/jfb.70443)
Supplement: Supplementary file 1 [file JFB-109-1125-s001.docx]

| **Ecosystem** | **Number Released (N) in 3 tag codes** | **Recoveries (C)** | **Ratio (C/N)** | **Mean C/N on batches** | **Mean, SE C/N**  **on Replicates** |
| --- | --- | --- | --- | --- | --- |
| **1983** | | | | | |
| River | 11703 | 59 | 0.0050 | 0.0063 | 0.0007 |
|  | 11637 | 78 | 0.0067 |  |  |
|  | 11620 | 86 | 0.0074 |  |  |
| Estuary | 11668 | 86 | 0.0073 | 0.0079 | 0.0005 |
|  | 11930 | 108 | 0.0090 |  |  |
|  | 11756 | 89 | 0.0076 |  |  |
| Transition | 11680 | 28 | 0.0024 | 0.0021 | 0.0003 |
|  | 11582 | 29 | 0.0025 |  |  |
|  | 11645 | 15 | 0.0013 |  |  |
| Marine | 11668 | 15 | 0.0013 | 0.0014 | <0.000 |
|  | 11675 | 16 | 0.0014 |  |  |
|  | 11769 | 19 | 0.0016 |  |  |
| **Total** | 140333 | 628 |  |  |  |
| **1984** | | | | | |
| River | 11616 | 47 | 0.0040 | 0.0036 | 0.0004 |
|  | 11715 | 52 | 0.0027 |  |  |
|  | 11683 | 48 | 0.0041 |  |  |
| Estuary | 12379 | 76 | 0.0061 | 0.0053 | 0.0009 |
|  | 11804 | 39 | 0.0033 |  |  |
|  | 11673 | 75 | 0.0064 |  |  |
| Transition | 11904 | 14 | 0.0012 | 0.0019 | 0.0008 |
|  | 11724 | 10 | 0.0009 |  |  |
|  | 11700 | 41 | 0.0035 |  |  |
| Marine | 11760 | 16 | 0.0014 | 0.0014 | <0.0000 |
|  | 11735 | 18 | 0.0015 |  |  |
|  | 11698 | 16 | 0.0014 |  |  |
| **Total** | 141391 | 452 |  |  |  |
| **1985** | | | | | |
| River | 10165 | 59 | 0.0058 | 0.0071 | 0.0007 |
|  | 10860 | 90 | 0.0083 |  |  |
|  | 10367 | 77 | 0.0073 |  |  |
| Estuary | 10823 | 64 | 0.0059 | 0.0058 | 0.0004 |
|  | 10659 | 69 | 0.0065 |  |  |
|  | 10519 | 53 | 0.0050 |  |  |
| Transition | 10803 | 48 | 0.0044 | 0.0056 | 0.0012 |
|  | 10593 | 46 | 0.0043 |  |  |
|  | 10464 | 85 | 0.0081 |  |  |
| Marine | 10577 | 78 | 0.0074 | 0.0079 | 0.0004 |
|  | 10645 | 83 | 0.0078 |  |  |
|  | 10585 | 91 | 0.0086 |  |  |
| **Total** | 127060 | 843 |  |  |  |

**Supporting Information File C** – Table 1. Number of juvenile Chinook salmon marked with three unique replicate tag codes (N) to identify release location each year, and numbers of adults recovered from the fisheries and the spawning grounds (C). The ratio of numbers recovered to those released (C/N), with means and standard errors of the mean provide an estimate of survival. Overall survival among all treatments was 0.47%.
