# Supplementary material for: Historical case study of a transfer experiment that demonstrated the importance of the estuarine ecosystem for the survival of Chinook salmon (Oncorhynchus tshawytscha) at the Campbell River estuary, British Columbia, Canada
Source: J Fish Biol. 2026 May 20;109(2):1125–40. doi: 10.1111/jfb.70443 (PMC13521722; doi:10.1111/jfb.70443)
Supplement: Supplementary file 2 [file JFB-109-1125-s002.docx]

**Supporting Information File A.** - Information describing juvenile and adult recovery methods, and equipment, and fishery locations:

**Juvenile Chinook** were tracked for five months following release from a bucket transported by helicopter to four locations. Fish were caught using a 14.7 m beach seine with wings 4.9 m (1 cm stretched mesh), bunt 4.9 m (3 mm stretched mesh) and depth 1.5 m. The net was probably most effective when water depth was less than 1.5 m but likely caught many of the surface-schooling fry and smolts in deeper sites as well. The net was fitted with 15 m towing lines off the wings. Usually the net was pulled off the beach using a 5 m inflatable boat with a 50 hp outboard engine or by personnel using waders, depending on water depth. The distance the net was taken offshore depended on local conditions such as currents, bottom topography, and floating obstacles (wharves, kelp beds) and therefore ranged from 5 to 15 m. On average, the net was estimated to sample about 30 to 90 m^2^ of the water surface. Up to ten Chinook salmon marked with an adipose clip from each seine set were preserved in formalin for retrieval of their CWTs. Only rarely were more than 10 marked fish captured in a seine. Details on catches are given in Brown et al. (1987).

**Adult Chinook** were recovered in various fisheries and from spawning ground surveys and hatchery egg takes. At the time of our study, in Canadian fisheries there was a mandatory inspection of 20% of the landed catch from both net and troll fisheries. When coded wire nose tagged (CWT) Chinook salmon were encountered, as indicated by a missing adipose fin, the heads were removed by dockside monitors. In sports fisheries, the heads of marked fish were voluntarily submitted by anglers to depots around the coast and monitors may also recover heads from anglers. At the spawning grounds on the Campbell and Quinsam Rivers, heads were taken from adipose-clipped fish that have spawned naturally and from fish that returned to the Quinsam hatchery. CWTs from catch and escapement were decoded in a central laboratory.

- The Regional Mark Processing Center data base categorizes returns to rivers as Freshwater Escapement and includes Quinsam River hatchery fish recorded from other rivers. CWT fish were only produced at hatcheries at the time of our work. There were two relatively-nearby hatcheries that could have reported a very small number of our released fish (Weil et al., 2025). Both were about 100 km to the south of the Campbell River system on the Strait of Georgia. As most Quinsam Chinook salmon while leaving and returning orient from the north this is an area where few smolts or adult returns were recorded (see below, Table 1; Supporting Information File B). There were no hatcheries operating at the time to the north on Johnstone Strait. Quinsam River Chinook salmon show a mean straying rate of 1.2% using CWTs and 0.3 % using thermal marking (Weil et al., 2025). As our Freshwater Escapement was 264 fish (Table 2 – MS), straying to neighbouring hatcheries (Big Qualicum, Little Qualicum and the Puntledge) may be expected to be less than four fish during the three experimental years.
- These methods for recovering tags in catch and escapement were coordinated with fisheries agencies in Alaska and Washington by the Pacific States Marine Fisheries Commission which also maintains a recovered CWT database used by researchers since 1976. The data are stored in files in the Regional Mark Processing Center (<https://www.rmpc.org/>). The recovery fishery categories and locations are given below.
- Details on statistical methods (expansion factors, corrections, etc.) used by both US and Canadian agencies at the time of this study are given in Johnson (1990).

| State or Province | Gillnet and Seine net | Troll | Sports |
| --- | --- | --- | --- |
|  |  |  |  |
| Alaska (Southeast) | 23 | 63 | 24 |
|  |  |  |  |
| British Columbia |  |  |  |
| JS/GS | 52 | 0 | 0 |
| NBC | 37 | 24 | 0 |
| CBC | 39 | 25 | 1 |
| JS/GS/JdF | 0 | 1 | 17 |
| NCBC | 0 | 0 | 30 |
| NWVI | 0 | 1 | 1 |
| JdFE | 1 | 0 | 0 |
| FW | 0 | 0 | 1 |
|  |  |  |  |
| Washington |  |  |  |
| JdFE | 0 | 0 | 1 |

Table 1: Number of records where at least one Chinook salmon from the transfer experiment was caught in a Seine and Gillnet net fishery, troll fishery and sport fishery in various locations in Canada and the USA, as categorized in the Mark Recovery data base.

Abbreviations:

JS/GS: Johnstone Strait/Georgia Strait

NBC: North Coast British Columbia

CBC: Central Coast British Columbia

NCBC: North Central Coast British Columbia

FW: Freshwater

JS/GS/JdF: Johnstone Strait/Georgia Strait/Juan de Fuca Strait

NWVI: Northwest Vancouver Island

JdFE: Juan de Fuca Strait East

Literature Cited

Brown, T.J., McAllister, C.D. & Kotyk, M.S. (1987). A summary of the salmonid catch-data from Campbell River estuary and Discovery Passage for the years 1982-1986. Can. Data Rep. Fish. Aquat. Sci. 650. 99p.

Johnson, J.K. (1990). Regional overview of coded wire tagging of anadromous salmon and steelhead in northwest America. *In*: *American Fisheries Society Symposium* (Vol. 7, pp. 782-816).

Weil, J., Luedke, W., Healy, T.M., Withler, R.E., Brown, N.A.W., Bokvist, J., & Porszt, E. (2025). The Magnitude and Extent of Chinook Straying from Hatcheries in Southern British Columbia. Canadian Science Advisory Secretariat (CSAS) Research Document 2024/024 Pacific Region. https://www.dfo-mpo.gc.ca/csas-sccs/Publications/ResDocs-DocRech/2025/2025_024-eng.html
