# Supplementary material for: Historical case study of a transfer experiment that demonstrated the importance of the estuarine ecosystem for the survival of Chinook salmon (Oncorhynchus tshawytscha) at the Campbell River estuary, British Columbia, Canada
Source: J Fish Biol. 2026 May 20;109(2):1125–40. doi: 10.1111/jfb.70443 (PMC13521722; doi:10.1111/jfb.70443)
Supplement: Supplementary file 3 [file JFB-109-1125-s003.docx]

Supporting Information File D-1. View of helicopter about to release experimental Chinook salmon smolts at Deepwater Bay, the marine ecosystem site (1983)

Supporting Information File D-2 a

Supporting Information File D-2 b

Supporting Information File D-2 a and b. Habitat development in the Campbell River estuary. a) artificial island/peninsula constructed with dredged gravel, before sedge transplant in 1983 b) portion of same island in 1985.
