# Supplementary material for: Historical case study of a transfer experiment that demonstrated the importance of the estuarine ecosystem for the survival of Chinook salmon (Oncorhynchus tshawytscha) at the Campbell River estuary, British Columbia, Canada
Source: J Fish Biol. 2026 May 20;109(2):1125–40. doi: 10.1111/jfb.70443 (PMC13521722; doi:10.1111/jfb.70443)
Supplement: Supplementary file 4 [file JFB-109-1125-s004.docx]

| **Release Ecosystem** | **Habitat** | **Number of sample sites** |
| --- | --- | --- |
| Estuary | Riparian/ sand | 1 |
|  | Natural marsh | 4 |
|  | Transplanted marsh | 2 |
|  | Gravel | 2 |
|  | Eelgrass in channel | 1 |
|  | Rip-rap revetment | 1 |
| **Total** |  | **11** |
| Transition | Gravel | 4 |
|  | Eelgrass in channel | 1 |
|  | Sand/Gravel | 1 |
|  | Gravel/cobble | 1 |
| **Total** |  | **7** |
| Marine | Sand/mud | 1 |
|  | Eelgrass | 5 |
|  | Kelp bed | 5 |
|  | Cobble | 2 |
|  | Sand/boulders | 1 |
|  | Gravel | 2 |
|  | Gravel/sand | 1 |
| **Total** |  | **17** |

**Supporting Information File D** - Table 1: Number of sample sites characterized by various habitat types at the three ecosystems where released marked smolts were recaptured in residency estimate study. Not all estuary sites are shown on Figure 1 because some sites overlapped or are off the image at the scale shown. From Brown et al., 1987.

Literature Cited:

Brown, T.J., McAllister, C.D. & Kotyk, M.S. (1987). A summary of the salmonid catch-data from Campbell River estuary and Discovery Passage for the years 1982-1986. Can. Data Rep. Fish. Aquat. Sci. 650. 99p.
