# Supplementary material for: Historical case study of a transfer experiment that demonstrated the importance of the estuarine ecosystem for the survival of Chinook salmon (Oncorhynchus tshawytscha) at the Campbell River estuary, British Columbia, Canada
Source: J Fish Biol. 2026 May 20;109(2):1125–40. doi: 10.1111/jfb.70443 (PMC13521722; doi:10.1111/jfb.70443)
Supplement: Supplementary file 5 [file JFB-109-1125-s005.docx]

| Release Year | Release ecosystem  Age and Year recovered | River | Estuary | Transition | Marine | Total n |
| --- | --- | --- | --- | --- | --- | --- |
| 1983 | 1984 - 2 y | 19 | 20 | 5 | 6 | 50 |
|  | 1985 – 3 y | 30 | 76 | 18 | 6 | 130 |
|  | 1986 – 4 y | 117 | 105 | 46 | 33 | 301 |
|  | 1987 – 5 y | 55 | 82 | 3 | 5 | 145 |
|  | 1988 – 6 y | 2 | 0 | 0 | 0 | 2 |
| 1984 | 1985 - 2 y | 16 | 31 | 4 | 1 | 52 |
|  | 1986 – 3 y | 34 | 74 | 23 | 6 | 137 |
|  | 1987 – 4 y | 45 | 52 | 17 | 42 | 156 |
|  | 1988 – 5 y | 32 | 33 | 14 | 1 | 80 |
|  | 1989 – 6 y | 0 | 0 | 7 | 0 | 7 |
| 1985 | 1986 - 2 y | 31 | 21 | 31 | 32 | 115 |
|  | 1987 – 3 y | 74 | 65 | 47 | 92 | 278 |
|  | 1988 – 4 y | 84 | 57 | 74 | 98 | 313 |
|  | 1989 – 5 y | 37 | 42 | 27 | 30 | 136 |
|  | 1990 – 6 y | 0 | 1 | 0 | 0 | 1 |

**Supporting Information File E** – Table 1: Age distribution of recoveries from releases to each of the four ecosystems, each year. Fish were recovered in the fishery and from escapements to the natural spawning grounds and the hatchery from 1983 to 1990. These data are the basis for Figure 3 in the MS and Supporting Information Figure 1, below.

**Supporting Information File E** – Figure 1: Age frequency of recoveries by release location each year. Release location had an influence on age of recovery; river and estuary releases were older in 1983 and younger in 1984, and marine release recoveries were primarily four years old (chi X^2^, P<0.05; Figure 3 - MS). Age and release location were not associated in 1985 (P>0.05).
