# Supplementary material for: Historical case study of a transfer experiment that demonstrated the importance of the estuarine ecosystem for the survival of Chinook salmon (Oncorhynchus tshawytscha) at the Campbell River estuary, British Columbia, Canada
Source: J Fish Biol. 2026 May 20;109(2):1125–40. doi: 10.1111/jfb.70443 (PMC13521722; doi:10.1111/jfb.70443)
Supplement: Supplementary file 6 [file JFB-109-1125-s006.docx]

**Supporting Information File B**: Background on marine rearing areas of Campbell River Chinook salmon

The main areas where Campbell River Chinook salmon grow and become available to fisheries are the coastal regions of northern British and southeast Alaska (e.g. Hamilton and Buell, 1977; Weitkamp, 2010) as confirmed by the analyses of the partial data set of our adult return catch area information (Levings et al., 1989), preliminary analyses of the complete data set and Supporting Information File A -Table 1 documenting Mark Recovery Program fishery areas.

The juvenile salmon migrate northwest from the Discovery Passage/southern Johnstone Strait area, gradually grow larger and become more dependent on deeper water and pelagic ecosystems and hence show reduced catchability to beach seining. For example, beach seining in the northern Johnstone Strait region showed only “incidental” catches of Chinook salmon smolts (Johnson et al., 2018). However, Quinsam hatchery Chinook have been found in surface trawls over deeper water of the inshore and fjord areas of the northern Discovery Islands area. A few Quinsam hatchery Chinook salmon were found in two boat trawls in June and July ( 1982-13 fish, 1983 – 3 fish; Levings and Kotyk, 1983). Most were caught at sites north of or near Seymour Narrows. In later work only a small number of Quinsam hatchery Chinook salmon (3 out of total catch of 63 marked fish) were caught in surface trawls in Discovery Passage and adjoining Bute Inlet (Neville et al., 2025).

Very few Quinsam hatchery smolts are caught in the Strait of Georgia to the south of our study area and those that do are from the north end of the Strait adjacent to Discovery Passage. During a recent synthesis of survival data of Chinook salmon stocks known to rear in the Strait of Georgia data from six stocks were noted, none of them Quinsam (Pearsall et al., 2021). Furthermore, based on the results of the June and July deep-water tow-net surveys (1998-2024) in the Strait of Georgia, Quinsam hatchery Chinook salmon do not rear in this area (e.g. <0.01% of about 300 marked fish) (Neville et al., 2024 a, b, c).

Literature Cited:

Johnson, B.T., Gan, J.C.L, Janusson, C.V. and Hunt, B.P.V. (2018). Juvenile salmon migration dynamics in the Discovery Islands and Johnstone Strait; 2015–2017. NPAFC Doc. 1790. 10 pp. Hakai Institute, Institute for the Oceans and Fisheries and Department of Earth, Ocean and Atmospheric Sciences, University of British Columbia (Available at <http://www.npafc.org>).

Levings, C. D., & Kotyk, M. S. (1983). Results of two boat trawling for juvenile salmonids in Discovery Passage and nearby channels, northern Strait of Georgia. Can. Man. Rep. Fish. Aquat. Sci. 1730: ix + 55 p.Neville, C.M. (2024a). Juvenile Pacific Salmon Survey in the Strait of Georgia and Associated Waters, June 24 to July 9, 2015 (SOG Survey 2015-06). Can. Data Rep. Fish. Aquat. Sci. 1398: ix + 303 p.

Neville, C.M. (2024b). Juvenile Pacific Salmon Survey in the Strait of Georgia and Associate Waters, June 19 to July 4, 2017 (SOG Survey 2017-04). Can. Data Rep. Fish. Aquat. Sci. 1399: ix + 312 p.

Neville, C.M. (2024c). Juvenile Pacific Salmon Survey in the Strait of Georgia and Associated Waters, July 5 to 14, 2016 (SOG Survey 2016-12). Can. Data Rep. Fish. Aquat. Sci. 1400: ix +312 p.

Neville, C. M., Spencer, S., & Kevin, M. (2025). *Juvenile Pacific Salmon Survey in the Strait of Georgia and Associated Waters, June 21 to July 4, 2018 (SOG Survey 2018-03)* . Can. Data Rep. Fish. and Aquat. Sci. 1432: 289 p.

Pearsall I, Schmidt M, Kemp I, Riddell B. (2021). Factors limiting survival of juvenile Chinook salmon, coho salmon and steelhead in the Salish Sea: Synthesis of the findings of the Salish Sea Marine Survival Project. 2021: 142 <https://www.marinescience.ca/wp-content/uploads/2021/06/2021PSF-SynthesisPaper-Screen.pdf>

Weitkamp, L. A. (2010). Marine distributions of Chinook salmon from the west coast of North America determined by coded wire tag recoveries. *Transactions of the American Fisheries Society*, *139*(1), 147-170.
